# Pharmacophore-guided discovery of CDC25 inhibitors causing cell cycle arrest and tumor regression

**DOI:** 10.1038/s41598-019-38579-7

**Published:** 2019-02-04

**Authors:** Zeynep Kabakci, Simon Käppeli, Claudio Cantù, Lasse D. Jensen, Christiane König, Janine Toggweiler, Christian Gentili, Giovanni Ribaudo, Giuseppe Zagotto, Konrad Basler, Lorenzo A. Pinna, Giorgio Cozza, Stefano Ferrari

**Affiliations:** 1 Institute of Molecular Cancer Research, University of Zurich, Winterthurerstrasse 190, CH-8057 Zurich, Switzerland; 2 Department of Clinical and Experimental Medicine, Campus US, University of Linköping, S-58183 Linköping, Sweden; 3 Wallenberg Centre for Molecular Medicine, University of Linköping, S-58183 Linköping, Sweden; 4 Institute of Molecular Life Sciences, University of Zurich, Winterthurerstrasse 190, CH-8057 Zurich, Switzerland; 5 Department of Medical and Health Sciences, Campus US, University of Linköping, S-58183 Linköping, Sweden; 6 Department of Pharmacology, University of Padua, Via U. Bassi 58/B, I-35131 Padua, Italy; 7 Department of Biomedical Sciences, University of Padua, Via U. Bassi 58/B, I-35131 Padua, Italy; 8 Department of Molecular Medicine, University of Padua, Via U. Bassi 58/B, I-35131 Padua, Italy

## Abstract

CDC25 phosphatases play a key role in cell cycle transitions and are important targets for cancer therapy. Here, we set out to discover novel CDC25 inhibitors. Using a combination of computational methods, we defined a minimal common pharmacophore in established CDC25 inhibitors and performed virtual screening of a proprietary library. Based on the availability of crystal structures for CDC25A and CDC25B, we implemented a molecular docking strategy and carried out hit expansion/optimization. Enzymatic assays revealed that naphthoquinone scaffolds were the most promising CDC25 inhibitors among selected hits. At the molecular level, the compounds acted through a mixed-type mechanism of inhibition of phosphatase activity, involving reversible oxidation of cysteine residues. In 2D cell cultures, the compounds caused arrest of the cell cycle at the G1/S or at the G2/M transition. Mitotic markers analysis and time-lapse microscopy confirmed that CDK1 activity was impaired and that mitotic arrest was followed by death. Finally, the compounds induced differentiation, accompanied by decreased stemness properties, in intestinal crypt stem cell-derived *Apc/K-Ras*-mutant mouse organoids, and led to tumor regression and reduction of metastatic potential in zebrafish embryo xenografts used as *in vivo* model.

## Introduction

The Cell Division Cycle 25 family encompasses three highly conserved members of dual specificity phosphatases that specifically target Cyclin-Dependent Kinases (CDKs), acting as dose-dependent inducers of cell cycle transitions^1,2^. CDC25A primarily activates CDK2/CycE and CDK2/CycA at the G1/S transition and in S-phase^3^, though it also cooperates with CDC25B at the onset of mitosis^4^. CDC25B initiates CDK1/CycB activation at centrosomes during the G2/M transition^4,5^ and CDC25C causes full activation of CDK1 at mitotic entry^6^.

Genetic studies showed that thermosensitive *cdc25* yeast mutants could be reversibly arrested in the cell cycle^7^, providing the first demonstration of a regulatory role for CDC25. The mouse *Cdc25A* gene was shown to be the only family member endowed with an essential function during embryonic development^8^.

Overexpression of CDC25, particularly CDC25A and CDC25B, has been observed in a variety of human cancers and correlates with poor clinical prognosis^9^. Interestingly, although CDC25A overexpression alone is insufficient to drive tumor initiation, *CDC25A* has a clear role as rate-limiting oncogene in transformation by *RAS*^10^. Furthermore, point mutations in CDC25C have a critical role in the pathology of acute myelogenous leukemia (AML)^11^.

Since the identification of vitamin K as potent CDC25 inhibitor, compounds based on the structure of vitamin K, as well as on other structures, have been developed as CDC25 inhibitors^12–19^. However, most compounds failed to keep up with expectations, either due to rapid metabolism in tumor-bearing SCID mice^20^ or for not completing clinical trials^21^, and have thus not attained approval.

In light of the recent discovery that CDC25 is the therapeutic target of choice in triple-negative breast cancers, namely those that are negative for estrogen-, progesterone- and HER2-receptor expression and that are unresponsive to standard therapy^22^, we set out to develop novel CDC25 inhibitors. To this end, we conducted a pharmacophore-guided drug discovery program that led to the identification of scaffolds of the naphthoquinone group displaying inhibition of CDC25 in enzymatic assays. In cultured cells, the most potent compounds induced inhibition of CDK1 activity and function, with block of mitotic transition followed by cell death. In mouse *Apc/K-Ras* mutant duodenal organoids, low doses of CDC25 inhibitors caused arrest of proliferation and expression of differentiation markers, whereas high doses induced cell death. In zebrafish embryos, used as *in vivo* xenograft model, the CDC25 inhibitors led to tumor regression and reduction of metastases.

## Results

### Pharmacophore-guided library screening and hit selection

**Figure 1:**
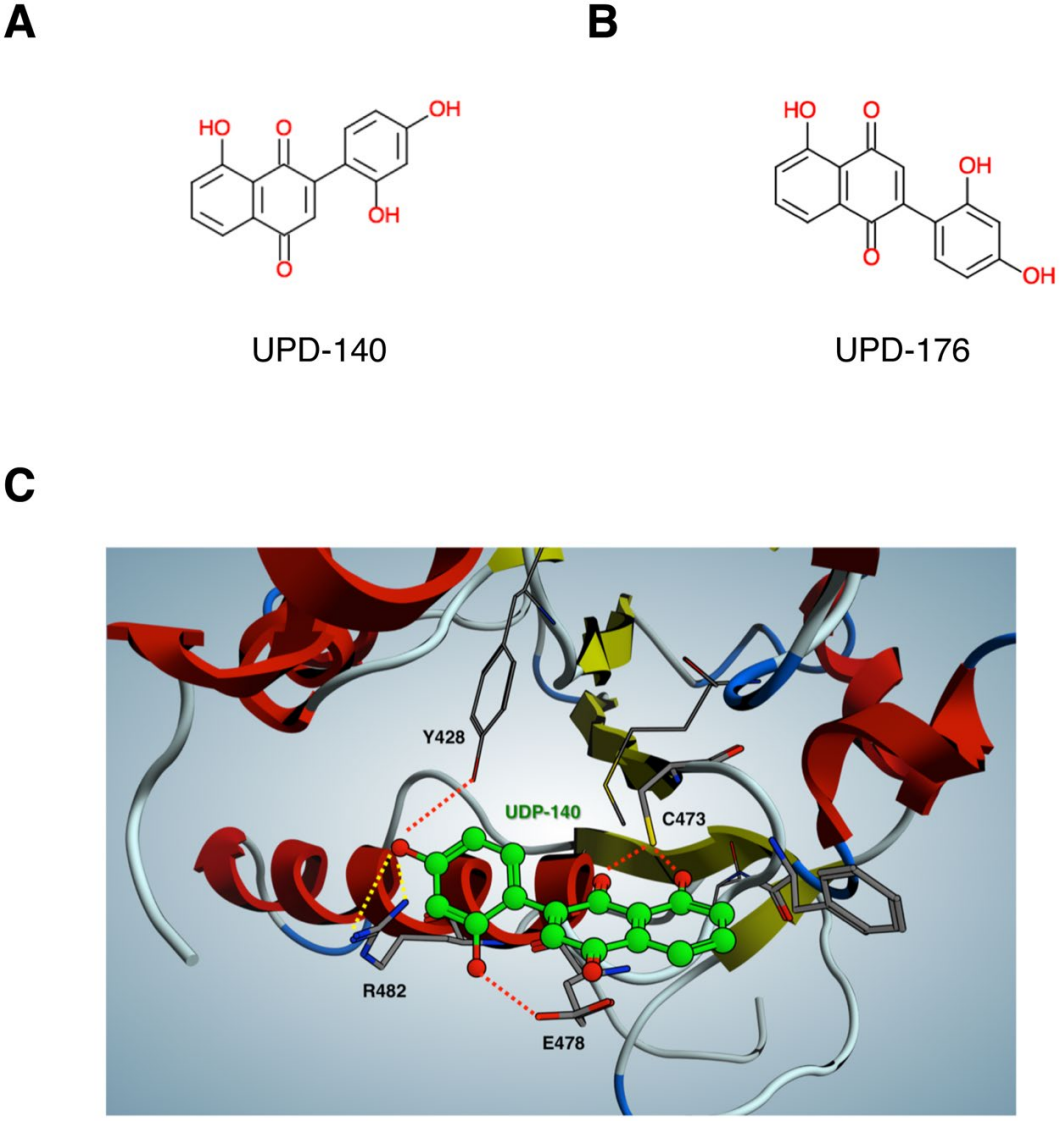
Structure and docking of CDC25 inhibitors. (**A,B**) Structure of 2-(2′,4′-dihydroxyphenyl)-8-hydroxy-1,4-naphthoquinone (UPD-140) and 5-hydroxy-2-(2,4-dihydroxyphenyl)naphthalene-1,4-dione (UPD-176). (**C**) Molecular docking of UPD-140 into CDC25B catalytic site.

To the end of retrieving novel CDC25 inhibitors from an *in silico* virtual library that was built from a proprietary database of synthetic molecules, we implemented a number of computational strategies (Fig. S1A), according to established protocols^23^. First, CDC25 inhibitors belonging to three classes - natural products, quinones and electrophiles^24^ - were subjected to a linear fragmentation process^25^ implemented in MOE Suite^26^, in which input structures were split into small pieces by removing the least “scaffold-like” extremity until indivisible essential fragments were obtained. Next, the molecular entities returned by this process, ordered by increasing size, were used to build a series of pharmacophore models (Fig. S1B). The latter were optimized until the achievement of a final model, representative of the chemical features of scaffolds obtained from the fragmentation process. Finally, this model was used to examine a proprietary library through a pharmacophore-guided virtual screening process (MOE Suite). Compounds obtained from the first round of hit selection and belonging to different molecular families were tested at fixed concentration on recombinant CDC25A (Table S1). Reference compound in all tests was the established CDC25 inhibitor NSC-663284, a para-quinonoid derivative of vitamin K^27^. Naphthoquinones UPD-140 (Fig. 1A, 2-(2′,4′-dihydroxyphenyl)-8-hydroxy-1,4-naphthoquinone) and UPD-176 (Fig. 1B, 5-hydroxy-2-(2,4-dihydroxyphenyl)naphthalene-1,4-dione) appeared to be the most effective inhibitors of CDC25 phosphatase activity. Based on the structure of UPD-140 and UPD-176, and exploiting the crystal structure of CDC25B^28^, along with available homology models for CDC25A and CDC25C, we performed hit expansion/optimization through a molecular docking strategy (Fig. 1C). Identification of pockets and surface sites through the localization of regions of tight atomic packing suggested two close cavities suitable to accommodate the compounds. Both cavities are highly conserved in the three enzymes and one is superimposable with the phosphatase catalytic site (Table 1). Starting point for prioritization of scaffolds was the presence of a quinone moiety, which appeared to be a necessary condition for optimal anchoring of compounds in CDC25 catalytic pocket. Reassessment of the library, based on the structure of UPD-140 and UPD-176, followed by i*n vitro* enzymatic assays revealed eight additional compounds as effective inhibitors of CDC25 phosphatase activity, all of them being 1,4-naphthoquinones with hydroxyl groups either in position 5 or 8 (Table S2).

**Table 1:** Residues lining the two cavities of CDC25 retrieved by the Site Finder approach.

Cavity 1			Cavity 2		
CDC25A	CDC25B	CDC25C	CDC25A	CDC25B	CDC25C
Tyr 387	Tyr 428	Tyr 332	Asp 336	Glu 377	His 281
*Cys 431*	*Cys 473*	*Cys 377*	Ile 338	Ile 379	Ile 283
Phe 433	Phe 475	Phe 379	Gly 339	Gly 380	Gly 284
Ser 434	Ser 476	Ser 380	Tyr 345	Phe 386	Cys 290
Glu 436	Glu 478	Glu 382	Asp 356	Asp 397	Asp 301
Gly 438	Gly 480	Gly 384	Leu 357	Leu 398	Leu 302
Arg 440	Arg 482	Arg 386	Lys 358	Lys 399	Lys 303
Met 489	Met 531	Met 435	Cys 442	Cys 484	Cys 388
			Arg 446	Arg 488	Arg 392
			Arg 450	Arg 492	Arg 396
			Glu 462	Glu 504	Glu 408
			Leu 463	Met 505	Leu 409

The catalytic cysteine in cavity 1 of each phosphatase is shown in italics.

### Enzymatic characterization and profiling of CDC25 inhibitors

**Figure 2:**
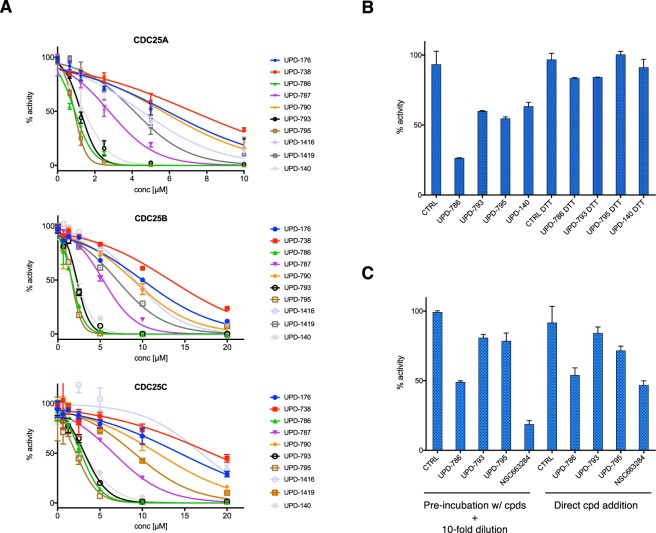
Enzymatic analysis of CDC25 inhibitors. (**A**) Dose-response studies with the indicated compounds on recombinant CDC25A, CDC25B and CDC25C. (**B**) CDC25A was treated in the presence or the absence of the reducing agent DTT (10 mM) prior to incubation with the compounds at a concentration proximal to their IC_50_ (1.25 μM). (**C**) Left: CDC25A was treated with the indicated compounds at a concentration ~3x IC_50_ (5 μM for UPDs and 2 µM for NSC663284), diluted 10-fold and assayed for phosphatase activity at the remaining compound concentration (0.5 and 0.2 μM, respectively). Right: As comparison, CDC25A was assayed upon direct addition of compounds at 0.5 or 0.2 μM concentration, respectively.

Selected naphthoquinone-based inhibitors were examined in dose-response assays on CDC25 phosphatases. Compounds UPD-786, UPD-793, UPD-795 and UPD-140 appeared to be the most potent inhibitors, displaying IC_50_ of 0.89 μM, 0.92 μM, 1.25 μM and 1.42 μM on CDC25A that are comparable to the IC_50_ of NSC-663284 (0.38 μM) (Figs 2A and S2A)^29^. A similar pattern of inhibition was observed on CDC25B and CDC25C, though with slightly higher IC_50_ values (Fig. 2A). Kinetic analysis conducted with UPD-795 revealed a mixed mechanism of enzyme inhibition, as indicated upon data analysis with the mixed-model inhibition equation^30^ (Table S3 and Fig. S2B), confirming the predictions of docking studies.

Considering that quinones are oxidizing agents, known for their ability to generate reactive oxygen species (ROS) in biological systems, and that the Cys residue in CDC25 active site is highly susceptible to oxidation^31^, we decided to assess a possible role for redox reactions in the inhibition of CDC25 by the most potent naphthoquinones described here. To this end, we treated CDC25A with an excess of the reducing agent DTT before addition of the compounds at a concentration proximal to their IC_50_. The data indicated that the presence of DTT in the reaction prevented inhibition of CDC25 (Fig. 2B).

Furthermore, since the quinone scaffolds so far described in the literature are in part reversible and in part irreversible CDC25 inhibitors, we decided to assess the mode of action of our most potent naphthoquinone inhibitors. To this end, we pre-incubated CDC25A with excess compound (5 μM, corresponding to ~3x IC_50_) and subsequently assayed for remaining phosphatase activity upon 10-fold dilution of the pre-incubation mix (hence at 0.5 μM compound). The data showed that the enzyme activity remaining upon dilution of the pre-incubation mix was comparable to the activity detected upon direct treatment of the phosphatase with 0.5 μM compounds, indicating a reversible mode of action (Fig. 2C). This was not the case for NSC-663284, which is an established irreversible inhibitor of CDC25 (Fig. 2C).

To assess specificity, we profiled two of the most potent CDC25 inhibitors, UPD-795 and UPD-140, against a panel of protein phosphatases. The data revealed that, when tested at concentrations close to the IC_50_ for CDC25A, both compounds also inhibited human PP5 activity to about 50% (Table S4), whereas none of the other phosphatases examined was affected.

### Cellular characterization of CDC25 inhibitors

**Figure 3:**
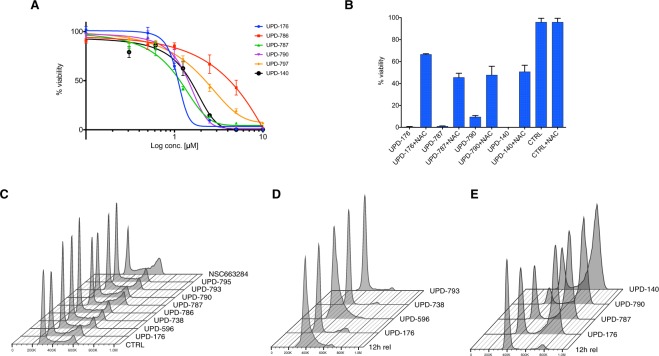
CDC25 inhibitors affect cell viability and the cell cycle. (**A**) HeLa cells were treated with increasing amounts of compounds and cell viability was determined. (**B**) Cells were treated in the presence or the absence of NAC before administration of compounds at doses ~3x IC_50_ (5 μM) and cell viability was determined. (**C**) Cells were treated with the indicated compounds (10 μM, 15 h). NSC663284 was used as comparison (5 µM, 15 h). DNA was stained with DAPI. (**D**) HeLa cells were synchronized at G2/M by treatment with Thymidine (1 mM, 15 h), release in full medium followed by addition of the CDK1 inhibitor Ro-3306 (9 μM, 15 h). Five hours upon release from Ro-3306, the indicated compounds were added (10 μM) and cells were harvested and analyzed 12 h upon release from Ro-3306. (**E**) HeLa cells were synchronized at G1/S by 2x thymidine block-release, compounds (10 μM) were added at 5 h and cells were analyzed at 12 h from release.

To assess CDC25 inhibitors in cells we performed viability assays on HeLa cells upon treatment with increasing compound doses. The data revealed IC_50_ of 1.08 μM, 1.41 μM, 0.90 μM and 1.20 μM for UPD-176, UPD-790, UPD-787 and UPD-140 respectively (Fig. 3A), all values being lower than that of the reference compound NSC-663284 (IC_50_ = 2.57 μM) (Fig. S3A). Compounds UPD-738 and UPD-786 appeared to be slightly less potent than NSC-663284, though both displayed IC_50_ values <10 μM (4.40 μM and 6.50 μM, respectively) (Fig. S3B).

To evaluate in a cellular context the oxidation-based mechanism of inhibition, we treated cells with the aminothiol N-Acetylcysteine, an antioxidant, prior to administration of the compounds. At compound doses exceeding 3x the IC_50_ (5 μM), cell viability was rescued to ~50% of controls in the presence of the reducing agent (Fig. 3B), hence confirming results obtained in biochemical assays.

To assess effects of the CDC25 inhibitors on cell cycle progression we administered compounds to HeLa cells and examined DNA content by flow cytometric analysis. In asynchronous HeLa cells, treatment with UPD-596, UPD-738, UPD-786, UPD-793 or UPD-795 caused an increase of the G1 population, indicating that G1/S was likely the point of action for these compounds. On the other hand, UPD-176, UPD-787 and UPD-790 were most effective in causing accumulation of cells at G2/M (Fig. 3C). Similar results were obtained in U2OS cells (Fig. S4). Since HeLa cells can be conveniently synchronized at specific points in the cell cycle (Fig. S5A), they were treated with UPD-176 or UPD-795 at the time of release from a double-thymidine block (early S-phase). Under these conditions, progression through S-phase was effectively held in check as compared to controls (Fig. S5B). To precisely assess effects of the compounds on the G1/S transition, HeLa cells were synchronized at G2/M, released to allow completion of mitosis and re-entry into the cell cycle, and treated in mid-G1 with UPD-176, UPD-596, UPD-738 or UPD-793. Analysis of cell cycle progression at 12 h post-release, a time when control cells entered S-phase, showed that UPD-176, UPD-738 and UPD-793 effectively blocked cells in G1, whereas UPD-596 appeared to be ineffective in this respect (Fig. 3D). To assess the effect of compounds on the G2/M transition, double-thymidine synchronized HeLa cells were released and treated with UPD-176, UPD-787, UPD-790 or UPD-140 5 h upon release, namely in late S-phase (Fig. S5A). Flow cytometric analysis of cell cycle progression at 12 h post-release, when control cells largely moved to the next G1 phase, showed that all compounds caused accretion of the G2/M peak, with UPD-790 apparently being the most effective of all (Fig. 3E). To exclude the possibility that the observed cell cycle arrest would be secondary to genotoxic effects, we examined the biomarker γ-H2AX at the onset of mitosis in cells treated with the compounds. The data showed that, in comparison to a potent genotoxic agent, no damage to DNA occurred upon treatment with the naphthoquinone compounds (Fig. S6).

**Figure 4:**
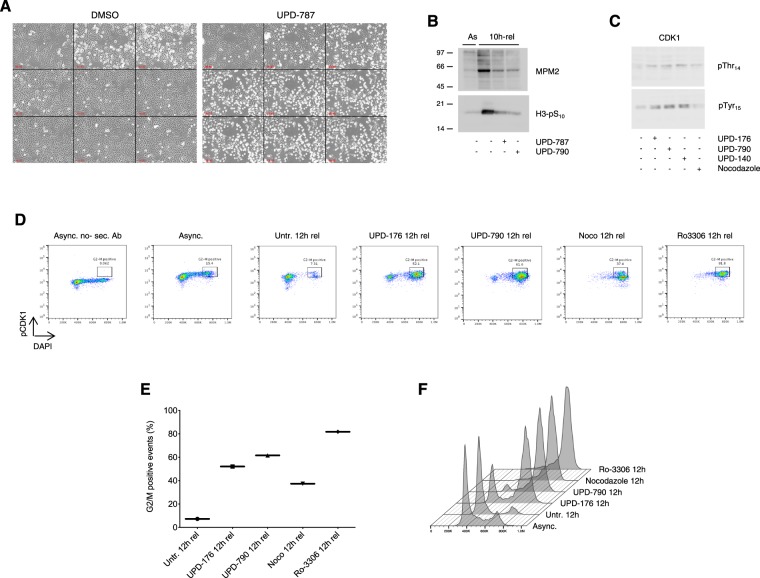
CDC25 inhibitors impair the execution of mitosis. (**A**) Phase contrast stills of HeLa cells synchronized by 2x thymidine block-release, treated with vehicle alone or UPD-787 (10 μM) 5 h upon release and visualized from 7.5 h to 21.5 h upon release (4 frames/h). (**B**) Western blot analysis of MPM2 epitopes and Histone H3 phosphorylation (pSer_10_) in HeLa cells treated as in (**A**) and examined at 10 h upon release from the 2x thymidine block. (**C**) Western blot analysis of CDK1 (pThr_14_ and Tyr_15_, respectively) from HeLa cells treated as in (**A**) and examined at 10 h upon release from the 2x thymidine block. (**D**) Flow cytometric analysis of CDK1-pTyr_15_ in cells treated as in (**A**) and examined 12 h upon release from the 2x thymidine block. Nocodazole (0.4 μg/ml) and Ro-3306 (9 μM) were used as controls for the level of CDK1 phosphorylation. Staining with secondary antibody only (left panel) was used to subtract self-fluorescence. (**E**) Quantification of positive events gated as shown in (**D**). (**F**) DNA content (DAPI staining) of the cells shown in D.

To visualize the execution of mitosis, we administered compounds to HeLa cells 5 h upon release from a double-thymidine block, and monitored cells by time-lapse microscopy for the subsequent 14 h. We observed that whereas control cells timely progressed through mitosis and moved to the next cycle, cells treated with UPD-787 could not complete mitotic transition and died before reaching G1 (Fig. 4A). A similar pattern was obtained with compounds UPD-790 and UPD-176 (Fig. S7A and data not shown). To better appreciate the response to inhibitors at the onset of mitosis, we administered low doses (5 μM) of UPD-787 or UPD-790 to synchronized HeLa Kyoto cells, which carry mCherry-H2B and GFP-α-tubulin. We observed a failed attempt to round up and execute mitosis, which was followed by membrane blebbing and death (Fig. S7A and Movies M1–M3). Western blot analysis revealed caspase-mediated cleavage of PARP-1, a signature of apoptosis, in cells treated with the compounds (Fig. S7B).

Biochemical analysis of mitotic markers at 10 h from the double-thymidine block-release showed decreased MPM2 epitopes in treated cells, a read out for CDK1 activity, and reduced Histone H3 phosphorylation at Ser_10_, a read-out for chromosome condensation (Fig. 4B). To directly assess cellular CDK1 activity under these conditions we used antibodies recognizing phosphorylated Thr_14_ or Tyr_15_, two sites in the P-loop of CDK1 catalytic domain, the phosphorylation of which hampers kinase activity and that are selectively dephosphorylated by CDC25^32,33^. As expected, at 12 h from the double-thymidine block-release point, control cells progressed to the G1 phase of the next cycle displaying low pThr_14_/pTyr_15_-CDK1, whereas cells treated with the compounds accumulated at G2/M (Fig. 4F) displaying high pThr_14_/pTyr_15_-CDK1 (Fig. 4C–E). The level of pTyr_15_-CDK1 in cells treated with CDC25 inhibitors appeared to be intermediate between that of cells treated with Ro-3306, a specific inhibitor of CDK1, and of cells treated with Nocodazole, an agent that interferes with tubulin polymerization causing arrest at pro-metaphase with active CDK1 (Fig. 4E).

### CDC25A overexpressing cell lines are sensitive to CDC25 inhibitors

**Figure 5:**
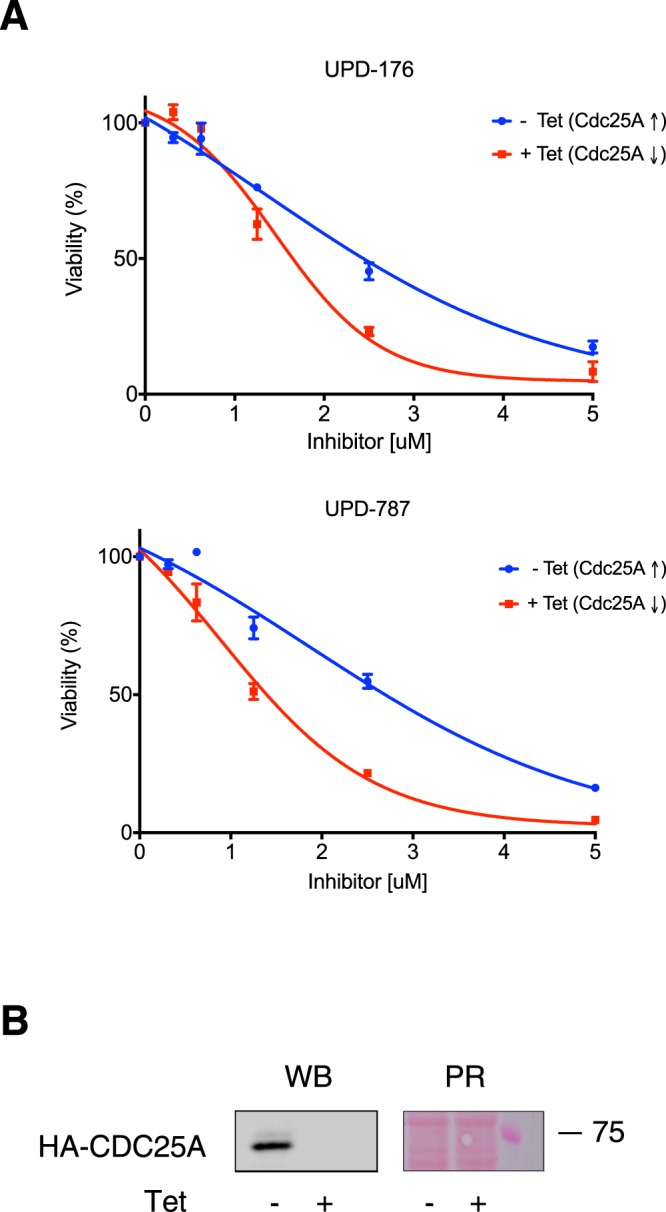
Cells overexpressing CDC25 are sensitive to CDC25 inhibitors. (**A**) U2OS Tet-OFF cells expressing HA-CDC25A were treated with the indicated compounds and cell viability was determined. P-values were determined by non-linear regression analysis and curve fitting comparison using the extra sum-of-squares F-test. (**B**) Western blot (WB) analysis of HA-CDC25A expression in the presence or absence of tetracycline. PR: Ponceau Red staining.

To determine whether cancer cells overexpressing CDC25 are sensitive to treatment with the compounds identified in this study, we initially selected cell lines that carry activating bi-allelic mutation of *K-Ras* (*G12S)* and are reported to express high level of CDC25B (http://www.proteinatlas.org/). However, Western blot analysis of cytoplasmic and nuclear extracts of such cell lines (A549 and Colo741) compared to HeLa, revealed lack of correspondence between the mRNA levels reported in databases and actual protein expression (Fig. S8). Similar results were obtained upon analysis of CDC25A and CDC25C protein expression (Fig. S8). Given the lack of an appropriate cell line model, we resorted to a system where CDC25A is expressed in a tetracycline-dependent manner and has been previously described^34^. Viability assays showed that cells grown in the absence of tetracycline, a condition inducing ectopic expression of HA-CDC25A to high extent, remained sensitive to treatment with UPD-176 or UPD-787 (Fig. 5A,B). P-values, determined by non-linear regression analysis of the data and curve fitting comparison, resulted to be < 0.0001 for both UPD-176 and UPD-787 (Fig. S9), indicating that the difference between tet-induced and non-induced cells was significant.

### CDC25 inhibitors arrest growth of *Apc/K-Ras* intestinal organoids

**Figure 6:**
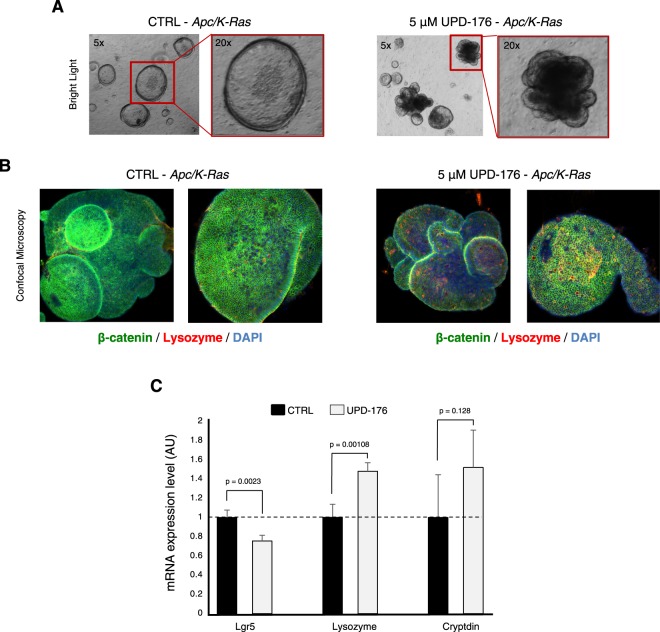
CDC25 inhibitors block the growth of K-Ras-dependent intestinal organoids. (**A**) Organoids reproducing duodenal crypts and derived from *Apc/K-Ras* mouse small intestine (left) were grown in the presence of UPD-176 (right). Phase contrast stills were obtained at 48 h of treatment. (**B**) Confocal immunofluorescence microscopy of *Apc/K-Ras* organoids treated as in A and stained for proliferation (β-catenin) or differentiation (lysozyme) markers. DNA was stained with DAPI. (**C**) qRT-PCR on cDNA obtained from control- and UPD-176-treated organoids was conducted using primers to the indicated markers.

Having established the effect of CDC25 inhibitors on the growth of cells in monolayer cultures, we addressed the ability of selected compounds to affect the growth of organoid cultures. Since CDC25A cooperates with, and is rate-limiting in tumorigenesis induced by *Ras*^10^, we generated and cultured 3D-organoids from stem cells of the intestinal crypts of *Apc/K-Ras* mice^35^. The latter carry the loss-of-function mutation *Apc1638N* combined with the *villinCre*-driven gain-of-function *K-RasG12V* mutation^36^. Upon seeding *Apc/K-Ras* organoids along with UPD-176 (5 μM), we observed that growing spheroids partially invaginated (Fig. 6A), indicating reduction of stem cell renewal accompanied by increased differentiation^37^, whereas higher doses of the compound caused massive death (data not shown). Consistent with the pattern of invagination at low compound treatment, immunofluorescence confocal imaging revealed augmented expression of lysozyme, a marker for cells exiting the proliferative compartment of the crypt and acquiring a differentiated state (Fig. 6B). Quantitative RT-PCR performed on the stemness marker *Lgr5* and on the differentiation markers Lysozyme and Cryptdin confirmed the confocal microscopy data (Fig. 6C).

### CDC25 inhibitors cause tumor regression and block the formation of metastasis in an *in vivo* model

**Figure 7:**
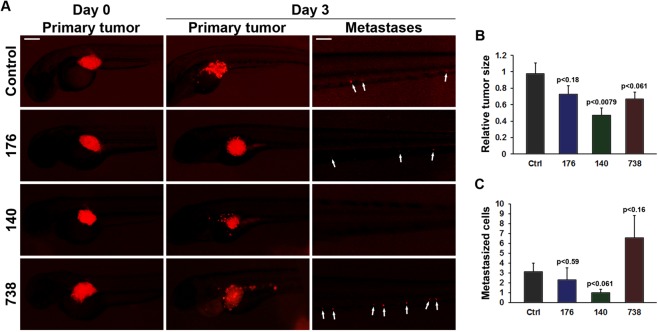
CDC25 inhibitors cause tumor regression and absence of metastasis in a zebrafish *K*-*Ras*-dependent tumor model. (**A**) Fluorescent microscopy images of HCT116 tumor xenografts (shown in red) immediately after implantation (Day 0) in two-days old zebrafish *larvae* and three days (Day 3) after implantation and treatment with DMSO vehicle (Control), UPD-176 (176), UPD-140 (140) or UPD-738 (738), in five-days old zebrafish *larvae*. Primary tumors and metastatic sites are shown separately. Metastasized tumor cells are indicated with white arrows. Size-bars in primary tumor images and metastatic site images correspond to 100 µm and 50 µm, respectively. (**B**,**C**) Quantifications of relative tumor volumes indicating the primary tumor size at day 3 relative to day 0 (**B**) or the number of metastasized tumor cells to the bone-marrow-like caudal hematopoietic plexus three days after tumor implantation (**C**). Results are shown as means (±SEM) of 17, 12, 12 and 17 embryos in the control, UPD-176, UPD-140 and UPD-738 groups, respectively. P-values indicate comparison between control and drug treated groups.

Next, we evaluated the effects of UPD-176, UPD-140 and UPD-738 on growth and metastatic potential of HCT116 tumors *in vivo* using a zebrafish xenograft model^38^. The compounds were well tolerated by the developing zebrafish embryos at the effective dose (10 µM) and did not cause signs of overt toxicity. Treatment of tumor-bearing fish *larvae* with UPD-140 for three days (from day 2 to day 5) caused a dramatic regression of the tumors, which were reduced to approximately half the size of the initial volume (p < 0.00784, Fig. 7A,B). The concurrent reduction in metastasis achieved borderline significance (p < 0.0604, Fig. 7A,C). Treatment with UPD-176 or UPD-738 displayed a tendency towards tumor regression, although not significant in comparison to vehicle (DMSO)-treated embryos (p < 0.1830 and p < 0.0609, respectively). For these compounds, also the variation of metastatic burden upon treatment with either compound did not appear to be significant (Fig. 7A–C). Taken together, these data demonstrate that inhibiting CDC25 may be an effective strategy to induce tumor regression and inhibit metastasis, *in vivo*.

## Discussion

Precision oncology is centered on the principle of continuous molecular interrogation of tumors and on the use of dedicated pharmacological tools to maximize success in therapy. Constant monitoring is essentially intended to assess resistance^39^ and reveal pathways to which tumors may become addicted during treatment^40^.

CDC25 phosphatases are overexpressed in a variety of human cancers^9,41^, are rate-limiting in tumorigenesis induced by *Ras*^10^, and were recently proposed as target of choice in unresponsive, triple-negative breast cancer^22^. In this study, we set out to identify novel CDC25 inhibitors. Taking advantage of knowledge gained in previous drug discovery programs^13,42^, we initially defined a pharmacophore that is common to compounds belonging to three distinct classes of established CDC25 inhibitors (Fig. S1). Performing a ligand-based virtual screen of a proprietary library, we identified naphthoquinone inhibitors of CDC25 phosphatases and conducted molecular docking studies on the crystal structure of CDC25B (Fig. 1, Tables S1 and S2). The phenyl moiety present in our scaffold represents a novel feature with respect to the chemical structure of previously described naphthoquinone inhibitors of the CDC25 family^14^. The interactions established by the R groups of the phenyl ring with residues located in the two cavities of CDC25 (Table 1) likely allow efficient anchoring of the naphthoquinone scaffold (Fig. 1), contributing to ameliorate potency in enzymatic and cellular assays. Biochemical studies on the identified compounds revealed a mixed mechanism of inhibition for all CDC25 members (Fig. S2), possibly indicating binding of the compounds to catalytic (Fig. 1C) and allosteric sites of the phosphatase, as also reported for other CDC25 inhibitors^18,19,27,43,44^. Mechanistically, we observed that compounds acted reversibly on CDC25 (Fig. 2C), likely through oxidation of the catalytic cysteine (Fig. 2B), a mode of action that we confirmed in cells (Fig. 3B). Such mechanism is compatible with a model proposed for CDC25 inhibitors^31^, according to which the thiolate group of the active-site cysteine undergoes very rapid conversion to sulfenic acid and it is protected from further conversion into irreversibly inactivated sulfinic acid by a back-door cysteine located in close proximity to the catalytic residue.

Cellular experiments showed that the most potent compounds arrested cells at the G1/S or the G2/M transition (Fig. 3D,E). Considering that these compounds displayed similar kinetics of inhibition on the three purified CDC25 isoforms (Fig. 2A), the reason for such preferential point of action in the cell cycle remains to be investigated. Interestingly, administration of compounds at the time of release from double-thymidine treatment showed minimal progression to S-phase at 6 hours of release (Fig. S5B). This may indicate that either turnover of the compound occurred during this time or, more likely, that the amount of CyclinE/CDK2 complex built up while cells accumulated at the point of forced arrest (i.e., early S-phase) was sufficient to support cell cycle progression despite inhibition of CDC25. The latter hypothesis is corroborated by the observation that when compounds were added in mid-G1, transition to S-phase was effectively blocked (Fig. 3D).

Since quinones undergo reduction to form semiquinone radicals or hydroquinones that, in turn, can react with oxygen to form superoxide radicals and/or hydrogen peroxide^45^, hence affecting a number of metabolic processes in the cell, we examined DNA damage induction as a possible side-effect relevant to the overall cellular response observed. Specifically, we quantified the biomarker γ-H2AX in synchronized cells that were treated with compounds. The data excluded the possibility that the cell cycle arrest observed in response to compound treatment could be secondary to genotoxic effects (Fig. S6).

To examine the cellular mechanism of action for the compounds described in this study, we focused on activation of CDK1 and entry into mitosis. Analysis of the extent of Thr_14_/Tyr_15_ phosphorylation, as indicator of kinase activation, and phosphorylation of CDK1 substrates, as indicator of kinase activity, revealed that the inhibitors effectively impaired both responses (Fig. 4B–E). Chromosome condensation normally occurring in prophase was also impaired in compound-treated cells, in line with flow cytometry data attesting a G2/M arrest under these conditions (Fig. 4B,F). Visual inspection of cells treated with the compounds showed that they could not execute mitosis, in line with our biochemical evidence, but rather underwent massive death (Fig. 4A). Administration of low concentration of the inhibitors to HeLa cells carrying mCherry-H2B allowed appreciating failed attempts to round up for mitosis, followed by membrane blebbing and death according to an apoptotic pattern (Fig. S7, Movies M2 and M3).

Enzymatic profiling of two of the most potent CDC25 inhibitors on a panel of phosphatases revealed PP5 as the only other target of the compounds (Table S4). PP5 controls a number of cellular processes including proliferation, migration and DNA damage. Interestingly however, PP5 activity is normally off due to folding of an N-terminal inhibitory domain onto the catalytic site, with ligand-mediated release of auto-inhibition occurring in response to cellular cues^46^. Hence, we argue that the specific G1/S and G2/M responses that we describe in this study are genuine effects of the identified compounds on CDC25 phosphatases.

Considering that CDC25A and CDC25B are overexpressed in a variety of human cancers^9^ and *CDC25A* was demonstrated to be a rate-limiting oncogene in transformation by *RAS*^10^, we examined the response of a cell line overexpressing CDC25A, as paradigm for the demonstration of compounds potency. The data confirmed that the compounds could effectively decrease viability of CDC25 overexpressing cells (Fig. 5).

Finally, we conducted studies in 3D-organoids, a system that reproduces architecture and function of the tissue of origin in reduced scale^47^ and in zebrafish as *in vivo* model of *K-Ras*-dependent tumors. Organoids obtained from stem cells of the intestinal crypts of *Apc/K-Ras* mice feature an internal cavity, corresponding to the crypt’s lumen, but lack the symmetry characteristic of this organ^48^. Fluorescence microscopy revealed decreased organoids size and acquisition of a differentiated state in response to CDC25 inhibitors, a pattern confirmed by qRT-PCR on selected markers (Fig. 6). These data are reminiscent of observations made on *Cdc25B*^*-/-*^*/Cdc25C*^*-/-*^ knock-out mice where *Cdc25A* was conditionally disrupted (*Cdc25A*^*fl-*^) in all tissues of the adult organism^8^. In this triple knock-out background, the authors reported large loss of the small intestine and crypts atrophy due to arrest at G1 and G2 phases of the cell cycle, which was paralleled by an increase of epithelial cell differentiation. As a whole, the triple knock-out studies and our data support the concept that blocking cell cycle progression through inhibition of CDC25 activity is beneficial to target tumor growth driven by mutant *Ras* and dependent on CDC25.

In zebrafish transparent embryos implanted with fluorescently labeled HCT116 cells, tumor growth/regression and metastasis can be accurately followed *in vivo*, over time, at single-cell resolution^38^. In this system, the efficacy of potential drug candidates can be accurately determined^49,50^. We obtained evidence that compounds bearing a pharmacophore common to NSC-663284, which was shown to have minimal antitumor activity in mice due to rapid metabolism^20^, are well tolerated and show clear anti-tumor efficacy in zebrafish xenograft models. Interestingly, compounds displaying the most potent effect in biochemical assays, showed the highest efficacy in the zebrafish model (Fig. 7), suggesting that these, and UPD-140 in particular, may be promising candidates for further investigation.

As a whole, the data reported in this study reveal the potential of novel naphthoquinone scaffolds acting as CDC25 inhibitors to target *Ras*-dependent tumors.

## Materials and Methods

### Chemistry

Commercially available chemicals were purchased from Sigma-Aldrich (Milan, Italy). For work-up and chromatographic purification, commercial grade solvents were used. Semi-preparative and preparative purifications of the synthesized compounds were carried out on Isolera One automated flash chromatography system (Biotage, Upsala, Sweden). The analytical profile of the synthesized molecules was in accordance with literature data (see below). ^1^H and ^13^C[^1^H] NMR spectra were recorded on a Bruker Avance III 400 MHz and a Bruker AMX 300 MHz spectrometers^51^. All spectra were recorded at room temperature. High-resolution mass spectra were recorded on an ESI-TOF Mariner from PerSeptive Biosystem (Stratford, Texas, USA), using electrospray ionization (ESI). Purity was assayed by HPLC, using a Varian Pro-Star system equipped with a Bio-Rad 1706 UV–VIS detector and an Agilent C-18 column (5 mm, 4.6 mm 150 mm). Water (A) and acetonitrile (B) were used as mobile phases with an overall flow rate of 1 mL/min and the following analytical method: 0 min (90% A–10% B), 15 min (10% A– 90% B), 20 min (10% A–90% B), 21 min (90% A–10% B), 255 min (90% A–10% B). Purity was over 97% (HPLC area).

#### *General procedure for the synthesis of UPD-596 (2-(2,3,4-trihydroxyphenyl)-1,4-naphthoquinone), UPD-597 (2-(2,4-dihydroxyphenyl)-1,4-naphthoquinone)*, *UPD-786 (8-hydroxy-2-(2,3,4 trihydroxyphenyl) naphthalene-1,4-dione), UPD-787 (8-hydroxy-2-(2,4-dihydroxy-3-methylphenyl) naphthalene-1,4-dione), UPD-788 (8-hydroxy-2-(2,4,6-trihydroxyphenyl)naphthalene-1,4-dione), UPD-790 (8-hydroxy-2-(2,4-dimethoxyphenyl)naphthalene-1,4-dione), UPD-140 (2-(2*′*,4*′*-dihydroxyphenyl)-8-hydroxy-1,4-naphthoquinone) and UPD-176 (5-hydroxy-2-(2,4-dihydroxyphenyl)naphthalene-1,4-dione)*

The preparation of the substituted naphthoquinones was performed according to previously reported procedures^51,52^. Briefly, the opportune naphthalene-1,4-dione (2 eq) and phenol (1 eq) were reacted in a mixture of acetic acid and 2 M H_2_SO_4_. After 2 h of stirring at room temperature under a nitrogen atmosphere, the reaction was stopped by the addition of water and neutralized with 5% sodium bicarbonate. The mixture was extracted with ethyl acetate, which was then evaporated to give the crude product. Purifications or separation of isomers were carried out by recrystallization or flash chromatography (n-hexane/ethyl acetate = 7:3 v/v).

#### General procedure for the synthesis of UPD-793 (3-(p-tolylthio)juglone) and UPD-795 (2-(3-methoxyphenylthio)-8-hydroxynaphthalene-1,4-dione)

The preparation of the sulfur-containing naphthoquinone derivatives was performed according to the previously reported procedure, with slight modifications^51^. Juglone (2 eq) and the opportune thiophenol (1 eq) were reacted in ethanol at room temperature. After 2 h of stirring at room temperature, the precipitate forming from the reaction mixture was filtered and recrystallized from ethanol to give the desired product.

#### General procedure for the synthesis of UPD-797 (8-hydroxy-2-(piperidin-1-yl)naphthalene-1,4-dione) and UPD-798 (N-(5-hydroxy-1,4-naphthoquinon-3-yl)morpholine)

The N-substituted naphthoquinones were prepared according to a previously reported procedure by reacting the precursor 3-Bromo juglone (1 eq) with a large excess of the appropriate amine (7 eq) in acetic acid. The work-up of the compounds was carried out following the reported procedure^51^.

#### Synthesis of UPD-724 (1,4-dihydroxy-6-anthraquinonecarboxylic acid)

The compound was synthesized from trimellitic anhydride and hydroquinone by a *Friedel Crafts* reaction (AlCl_3_, NaCl, 150 °C, 7 h) followed by a mild air oxidation, according to a previously reported procedure^53,54^.

#### Synthesis of UPD-738 (5-hydroxynaphtho-1,4-quinone)

5-hydroxynaphtho-1,4-quinone (juglone) was prepared according to a literature procedure^55,56^.

#### Synthesis of UPD-1416 (4-Hydroxy-2-methylquinoline-5,8-dione) and UPD-1419 (2-methyl-5,8-dihydro-5,8-dioxoquinoline)

UPD-1416 is a commercially available compound (Ark Pharm). UPD-1419 was prepared according to a previously reported procedure, allowing the preparation of 1,4-quinone derivatives starting from 1,4-dimethoxybenzenes using NBS^57^.

#### Synthesis of UPD-1382 (5,8-diaminonaphthalene-1,4-dione)

The compound was prepared according to the procedure reported in patent US2399355^58^.

### *In silico* protein preparation

The crystal structure of human CDC25A and CDC25B were retrieved from Protein Data Bank (PDB codes: 2Z5X and 1QB0 respectively) and processed in order to remove ligands and water molecules. Hydrogen atoms were added to the protein structures using standard geometries with the Molecular Operating Environment (MOE) software^26^. To minimize contacts between hydrogen atoms, the structures were subjected to Amber99 force-field minimization until the root mean square (rms) of conjugate gradient was < 0.1 kcal⋅mol^−1^⋅Å^−1^ (1 Å = 0.1 nm), keeping the heavy atoms fixed at their crystallographic positions. Human CDC25A and CDC25C were built using a homology modeling approach implemented into MOE^26^ with CDC25B as homologous template (PDB code: 1QB0). Sequence alignment was performed using the MOE Protein Align tool with BLOSUM62 as substitution matrix (CDC25A, 67% identity; CDC25C, 61% identity).

### Generation of virtual library and hit selection

An *in silico* virtual library was built from a proprietary database of synthetic molecules (2075 compounds) upon 2D to 3D conversion of the molecules chemical structure, optimization of compound conformers and addition of Gasteiger partial charges (MOE Suite). The strategy for hit selection is described in the Results section.

### *In silico* hit expansion/optimization

The structure of the first two hits, UPD-140 and UPD-176, provided a clue for the hit expansion/optimization phase through a Site Finder approach (MOE Suite), followed by a Molecular Docking protocol (Glide, Schrödinger Suite^59^). Site Finder allows the identification of pockets and surface sites by identifying regions of tight atomic packing using Alpha Shapes, a generalization of convex hulls developed in^60^ that has been successfully validated against several unrelated targets^61,62^. The procedure suggested two close cavities that are highly conserved in the three enzymes, one of them corresponding to the phosphatase catalytic site. Based on the Site Finder indications, a molecular docking process was performed using the Glide package XP procedure^63^ (Schrödinger Suite) and focusing on compounds featuring a quinone moiety.

### Microscopy

Cells were grown in 35 mm CellView™ cell culture dishes with glass bottom (# 627870, Greiner-BioOne) at a density of 2.5 × 10^5^ cells/ml in a humidified cell incubator maintained at 37 °C and 5% CO_2_. Cell were viewed by phase contrast microscopy with a 10x objective using an Olympus IX 81 motorized inverted microscope (Olympus, Hamburg, Germany) equipped with external temperature control chamber and CO_2_ bottle to maintain cells at 37 °C with 5% CO_2_. Transition through mitosis was documented by acquisition of four frames per hour over a period of 14 h using a CCD camera (Orca AG, Hamamatsu) and cellR^®^ software (Olympus). HeLa-Kyoto cells (mCherry-H2B/EGFP-α-tubulin) were visualized by fluorescence microscopy with an Olympus IX 81 microscope using a 10x objective and selecting Ex. = 492 ± 18 nm/Em. = 535 ± 50 nm for the green channel and Ex. = 572 ± 23 nm/Em. = 645 ± 75 nm for the red channel.

### Intestinal organoids culture

Intestinal crypts were isolated from *Apc*/*K-Ras* animals bearing *Apc*^*1638N*^ and villin-driven *K-Ras*^*G12V*^ alleles with slight modifications^36^ of a previously described method^64^. Briefly, crypts were isolated and purified, embedded in 50 μl matrigel drops (Corning, 356231) and overlaid with 500 μl organoid medium Advanced DMEM/F12 (Life Technologies, 11320-082) containing 2 mM GlutaMAX (Life Technologies, 35050-061), 10 mM HEPES buffer (Sigma, 83264-100ML-F), 0.5 U/ml Penicillin/Streptomycin (Life Technologies, 15070-063), N2 (Life Technologies, 17502-048), B27 (Life Technologies, 12587-010)]. Organoids were expanded for 5 days in growth factor supplemented medium containing 50 ng/ml mEGF (Life Technologies, PMG8041), 100 ng/ml mNoggin (Peprotech, 250–38) and 500 ng/ml hRSPO1 (R&D, 4645-RS-025). EGF and R-spondin were subsequently removed to select for organoids that had lost the wild-type *Apc* copy. The medium was changed every 2 days. Established mutant lines were passaged every four days by mechanical disruption with a bent P1000 pipette tip.

### *In vivo* tumor model

Implantation of HCT116 cells into zebrafish embryos were carried out essentially as previously described^38^. Briefly, HCT116 cells were cultured in McCoy’s medium supplemented with 10% FCS and penicillin/streptomycin until ~80% confluence, then labeled for 30 min at 37 °C in 6 μg/mL 1,1′-dioctadecyl-3,3,3′,3′-tetramethylindocarbocyanine perchlorate (DiI, Sigma) in PBS followed by washing 3x in PBS. Labeled cells were implanted in the perivitelline space of 48 hours post-fertilization (hpf) wildtype (AB) zebrafish embryos, kept from the 1-cell stage in 1-phenyl-2-thiourea (PTU)-containing E3-water. Approximately 200–500 cells were implanted in each embryo, which were then transferred to E3 medium containing PTU as well as 10 μM UPD-176, UPD-140 or UPD-738 appropriately diluted from stock solutions made in DMSO. As control, embryos were incubated in PTU-E3 with 0.1% DMSO (vehicle). Tumor-bearing embryos were incubated at 36 °C for three days and the change in tumor volume was evaluated as the size of the tumors at three days post-implantation (3 dpi) relative to the size immediately after implantation (0 dpi). Metastasis was evaluated at 3 dpi as the number of tumor cells present in the caudal hematopoietic plexus, the main metastatic site for tumors implanted in the perivitelline space.

## Supplementary information

Dataset 1

Movie M1 - Mitotic transition in control-treated Kyoto HeLa cells

Movie M1 - Mitotic transition in UPD-787-treated Kyoto HeLa cells

Movie M1 - Mitotic transition in UPD-790-treated Kyoto HeLa cells
