# Supplementary material for: Pharmacophore-guided discovery of CDC25 inhibitors causing cell cycle arrest and tumor regression
Source: Sci Rep. 2019 Feb 4;9:1335. doi: 10.1038/s41598-019-38579-7 (PMC6362118; doi:10.1038/s41598-019-38579-7)
Supplement: Supplementary file 1 — Dataset 1 [file 41598_2019_38579_MOESM1_ESM.pdf]

Pharmacophore-guided discovery of CDC25 inhibitors causing cell cycle arrest and tumor regression

Zeynep Kabakci[1]*, Simon Käppeli[1]*, Claudio Cantù[2,3,4]*, Lasse D. Jensen[5]*, Christiane König[1], Janine Toggweiler[4], Christian Gentili[1], Giovanni Ribaudo[6], Giuseppe Zagotto[6], Konrad Basler[4], Lorenzo A. Pinna[7], Giorgio Cozza[8,9] and Stefano Ferrari[1,9]

[1]Institute of Molecular Cancer Research, University of Zurich, Winterthurerstrasse 190, CH-8057 Zurich, Switzerland
[2]Department of Clinical and Experimental Medicine, Campus US, University of Linköping, S-58183 Linköping, Sweden
[3]Wallenberg Centre for Molecular Medicine, University of Linköping, S-58183 Linköping, Sweden
[4]Institute of Molecular Life Sciences, University of Zurich, Winterthurerstrasse 190, CH-8057 Zurich, Switzerland
[5]Department of Medical and Health Sciences, Campus US, University of Linköping, S-58183 Linköping, Sweden
[6]Department of Pharmacology, University of Padua, Via U. Bassi 58/B, I-35131 Padua, Italy
[7]Department of Biomedical Sciences, University of Padua, Via U. Bassi 58/B, I-35131 Padua, Italy
[8]Department of Molecular Medicine, University of Padua, Via U. Bassi 58/B, I-35131 Padua, Italy
[9] Corresponding Authors

SUPPLEMENTARY FILES

SUPPLEMENTARY MATERIALS AND METHODS

Antibodies and chemicals - The following antibodies were used for Western blot: Rabbit monoclonal antibodies to CDK1-pThr$_{14}$ (#2543**),** CDK1-pTyr$_{15}$ (#9111**),** Histone H3-pSer$_{10}$ (#9701) and CDC25B (#9525) were purchased from Cell Signaling Technology (Beverly, MA, USA). Mouse monoclonal antibodies to CDC25A (F-6, sc-7389), CDC25C (C-20, sc-327) and α-tubulin (YOL 1/34, sc-53030) were purchased from Santa Cruz Biotechnology (Santa Cruz, CA, USA). The mouse monoclonal antibody MPM2 (05-368) was from Upstate (Lake Placid, NY, USA). The mouse monoclonal antibody to the HA tag (HA.11, MMS-101P) was from BioLegend (S. Diego, CA, USA). The rabbit polyclonal antibody to PARP-1 (#227244) was obtained from Abcam. HRP-conjugated anti-mouse and anti-rabbit secondary antibodies were obtained from GE-Healthcare (Otelfingen, Switzerland). HRP-conjugated IgG-κ-BP was purchased from Santa Cruz Biotechnology (Santa Cruz, CA, USA).

The following antibodies were used for immunofluorescence on a Zeiss LSM 710 confocal microscope: mouse monoclonal to beta-catenin (Cl. 14, BD transduction Lab.), rabbit polyclonal to lysozyme (A0099, Dako). Secondary antibodies were: Alexa 488 goat anti-mouse, Alexa 555 goat anti-rabbit. Nuclei were visualized with 4',6-Diamidino-2-Phenylindole (DAPI, Sigma-Aldrich).

The CDK1 inhibitor Ro-3306 (Roche, Switzerland) was dissolved in DMSO at 9 mM stock concentration and stored in aliquots at -20 °C. Thymidine was obtained from SynGen Research Inc. (Sacramento, CA, USA) and dissolved in PBS as 200 mM stock solution before use. NSC-663284 (Sigma-Aldrich, Buchs, Switzerland) as well as all UPD compounds were dissolved in DMSO at 10 mM stock concentration and stored in aliquots at -20 °C.

Expression and purification of CDC25 phosphatases - CDC25A (kind gift of I. Hoffmann, Heidelberg, Germany), CDC25B and CDC25C (kind gift of B. Gabrielli, Queensland, Australia) in pGEX were expressed in *E. coli* BL21 cells and purified on 1 ml Glutathione Sepharose 4 Fast Flow columns as described [1].

Enzymatic assays - CDC25A, CDC25B and CDC25C were appropriately diluted in 10 mM Tris-HCl pH 8.0, 50 mM NaCl and assayed in 20 mM Tris-HCl pH 8.0, 75 mM NaCl, 0.5 mM EGTA, 1 mM DTT, 0.1 mg/ml BSA and defined concentrations of compounds. Reactions were started by addition of the substrate OMFP (0.1 mM). Assays were carried out at room temperature in duplicate or triplicate using transparent-bottom, V-shaped 96 well plates and read at defined intervals on a Molecular Devices SpectraMax microplate reader (ex: 490 nm / em: 525 nm). IC_{50} and kinetic parameters calculated from Lineweaver-Burk double-reciprocal plots of the data were obtained using GraphPad Prism software.

Cell culture - Wild-type HeLa and Kyoto HeLa cells stably expressing mCherry-H2B were maintained in DMEM+L-Glutamine (Gibco, Life Technologies, USA) supplemented with 5% fetal calf serum (FCS) (Gibco) and penicillin-streptomycin (100 U/ml - 100 μg/ml) (Gibco). A549 and Colo741 were grown and maintained like HeLa cells but with 10% FCS. HCT116 were maintained in McCoy's medium supplemented with 10% FCS and penicillin-streptomycin. U2OS-Tet-OFF cells expressing HA-CDC25 were grown and maintained in DMEM+L-Glutamine, 10% Tet-free FCS and penicillin-streptomycin in the presence or the absence of 4 μg/ml tetracycline (Sigma, T7660).

Cell synchronization - Cell synchronization was performed by double thymidine block-release protocol [2] or nocodazole treatment [3] and verified by flow cytometry.

Cell viability assays - Compounds viability upon 48h treatment with the compounds was assessed as described [4]. Data were plotted with Prism 7® and analyzed by non-linear regression. Comparison of single curve fitting to all data set within an experiment was performed with the extra sum-of-squares F-test to determine P values.

Western blotting - Cell lysis and immunoblot analysis were performed as previously described [5]. Proteins were revealed using the Western blotting detection kit WesternBright™ ECL (Advansta, Menlo Park, CA, USA) and signal imaged using the Fusion Solo system (Vilber Lourmat).

Flow Cytometry - To quantify DNA content cells were harvested, fixed with 70% ethanol (-20 °C) and stored overnight at +4 °C. Cells were washed with PBS, resuspended in PBS containing 0.1 mg/ml RNAse and DNA was stained with 1 µg/ml DAPI. To quantify phosphorylation of CDK1 at Tyr_{15} and H2AX at S_{139}, cells were harvested and examined as described [6]. Samples were measured on an Attune™ NxT Cytometer (ThermoFisher Scientific, Carlsbad, CA, USA) and data were analyzed with FlowJo® software v10 (FlowJo, LLC, Ashland, OR, USA).

Real Time qRT-PCR - Organoids were lysed in Tri-Reagent (Sigma) as previously described [7] and total RNA was isolated as described by manufacturer's instructions. For cDNA synthesis 1µg of total RNA was used and cDNA was synthesized by Transcription High Fidelity cDNA Synthesis kit (Roche). Real-time quantitative PCR reactions were performed in three independent biological replicates, each with technical triplicate, using the Applied Biosystems SYBR Green Kit and monitored by the ABI Prism 7900HT system (Applied Biosystem). The following primers were used:

Lgr5: Forward 5'-CTCCACACTTCGGACTCAACAG-3' and Reverse 5'-AACCAAGCTAAATGCACCGAAT-3'

Lysozyme: Forward 5'-CTGTGGGATCAATTGCAGTG-3' and Reverse 5'-GCGAGGAAGTGTGACCTCTC-3'

Cryptdin: Forward 5'-AGGAGCAGCCAGGAGAAG-3' and Reverse 5'-ATGTTCAGCGACAGCAGAG-3'

TATA-binding protein (TBP): Forward 5'-TTGACCTAAAGACCATTGCAC-3' and Reverse 5'-TTCTCATGATGACTGCAGCAAA-3'

Specificity of amplification products was verified considering the exponential amplification curves and the melting temperature analysis of the final amplification product.

Compound profiling - Selected compounds were profiled against a panel of protein phosphatases at Eurofins Pharma Discovery Services Ltd., Dundee, UK.

SUPPLEMENTARY REFERENCES

1 Hassepass, I. & Hoffmann, I. Assaying Cdc25 phosphatase activity. *Methods Mol Biol* **281**, 153-162, doi:10.1385/1-59259-811-0:153 (2004).

2 Krystyniak, A., Garcia-Echeverria, C., Prigent, C. & Ferrari, S. Inhibition of Aurora A in response to DNA damage. *Oncogene* **25**, 338-348, doi:1209056 [pii] 10.1038/sj.onc.1209056 (2006).

3 Ferrari, S. *et al.* Aurora-A site specificity: a study with synthetic peptide substrates. *Biochem J* **390**, 293-302 (2005).

4 Pierroz, V. *et al.* Dual mode of cell death upon the photo-irradiation of a RuII polypyridyl complex in interphase or mitosis. *Chemical Science* **7**, 6115-6124, doi:10.1039/C6SC00387G (2016).

5 El-Shemerly, M., Hess, D., Pyakurel, A. K., Moselhy, S. & Ferrari, S. ATR-dependent pathways control hEXO1 stability in response to stalled forks. *Nucleic Acids Res* **36**, 511-519, doi:gkm1052 [pii] 10.1093/nar/gkm1052 (2008).

6 Bologna, S. *et al.* Sumoylation regulates EXO1 stability and processing of DNA damage. *Cell Cycle* **14**, 2439-2450, doi:10.1080/15384101.2015.1060381 (2015).

7 Valenta, T. *et al.* Wnt Ligands Secreted by Subepithelial Mesenchymal Cells Are Essential for the Survival of Intestinal Stem Cells and Gut Homeostasis. *Cell Rep* **15**, 911-918, doi:10.1016/j.celrep.2016.03.088 (2016).

8 Copeland, R. A. Evaluation of enzyme inhibitors in drug discovery. A guide for medicinal chemists and pharmacologists. *Methods Biochem Anal* **46**, 1-265 (2005).

SUPPLEMENTARY FIGURE LEGENDS

Figure S1 - Flow chart for hit selection and example of linear fragmentation

(A) Flow diagram for the selection of CDC25 inhibitory compounds.

(B) Example of the linear fragmentation process for NSC-663284 and for indolyldihydroxyquinone, resulting in sets of molecular entities that were used to build a series of pharmacophore models.

Figure S2 - Compound comparison and kinetic parameters for UPD-795

(A) Magnification of the low concentration range for the compounds shown in Fig. 2A (left) in comparison with NSC-663284 (right). Dashed lines show the IC_{50} for the most potent compounds.

(B) CDC25A, CDC25B and CDC25C were assayed at appropriate dilution with increasing substrate concentrations (0-6.25-12.5-25-50-100-200 μM) in the presence of UPD-795 (0.625 μM:▲; 1.25 μM: ■; 2.5 μM: ●) or vehicle (:▼).

Figure S3 - Comparative cell viability assays

(A, B) HeLa cells were treated with increasing amounts of the indicated compounds in comparison to the reference compound NSC-663284 and cell viability was determined.

Figure S4 - Effect of selected compounds on U2OS cells

Flow cytometric analysis of non-synchronized U2OS cells treated with the indicated compounds (10 μM) for 15h.

Figure S5 - Cell cycle synchronization of HeLa cells and treatment with compounds

(A) Flow cytometric analysis of 2x thymidine block-released HeLa cells displaying timely cell cycle progression.

(B) Double-thymidine synchronized cells were left untreated or treated with UPD-176 or UPD-795 (10 μM) at the point of release and examined for the indicated times.

Figure S6 - Analysis DNA damage response

(A) Flow cytometric analysis of γH2AX induction by camptotechin (1 μM, 4h).
(B) Double-thymidine synchronized HeLa cells were treated with the indicated compounds (10 μM) 5h upon release from the block and examined at 10h.

Figure S7 - Effect of CDC25 inhibitors on mitosis

(A) Phase contrast stills of Kyoto HeLa cells (mCherry-H2B/EGFP-α-tubulin) synchronized by 2x thymidine block-release protocol and treated with vehicle alone (CTRL), UPD-787 (5 μM) or UPD-790 (5 μM) 5h upon release. Cells were visualized for 12h in a time-course fashion starting at 6h upon release from the 2x-thymidine block point and taking 4 frames per hour.
(B) Western blot analysis of PARP-1 cleavage using extracts of double-thymidine synchronized HeLa cells that were treated with the indicated compounds (10 μM) 5h upon release from the block and examined at 10h.

Figure S8 - CDC25 expression in cancer cell lines

Cytoplasmic (CE) and nuclear extracts (NE) of HeLa, A549 and Colo741 cells (50 μg) were resolved on 10% SDS-polyacrylamide gels and expression of CDC25A, CDC25B (B1 = 64987 Da; B2 = 60756 Da) and CDC25C was revealed with appropriate antibodies. Molecular weight markers are indicated. PR: Ponceau Red.

Figure S9 - Analysis of sensitivity to CDC25 inhibitors

Determination of P-values for the null-hypothesis (one curve fits all data) by non-linear regression analysis of the data and curve fitting comparison using the extra sum-of-squares F-test.

Figure S10 – Uncropped original Western blots for Figs. 4B, 4C, 5B and S8

SUPPLEMENTARY TABLES

Table S1 - Enzymatic assay on CDC25A with compounds of the first round of selection

The compound concentration used in the assays was 20 μM. NSC-663284 was used at concentration of 2 μM. The results are displayed as activity remaining (%) in the presence of compound relative to control reactions conducted in the presence of vehicle alone (DMSO). Values are averages of duplicate determinations ± standard deviations. One of two representative experiments is shown.

**Compound**	**Class**	% remaining activity ± SD
Ctrl	DMSO	100
NSC-663284	Quinolinedione	1.2 ± 0.9
UPD-22	Peptoid derivative	109.2 ± 3.3
UPD-80	Barbituric acid derivative	93.6 ± 6.6
UPD-86	Barbituric acid derivative	84.0 ± 1.1
UPD-88	Barbituric acid derivative	37.3 ± 1.1
UPD-93	Barbituric acid derivative	125.5 ± 2.1
UPD-140	Naphthoquinone	0.1 ± 0.1
UPD-151	Barbituric acid derivative	123.9 ± 6.0
UPD-155	Barbituric acid derivative	94.6 ± 3.1
UPD-166	Barbituric acid derivative	97.5 ± 3.6
UPD-168	Barbituric acid derivative	121.3 ± 3.2
UPD-170	Barbituric acid derivative	88.2 ± 11.3
UPD-172	Barbituric acid derivative	22.7 ± 2.7
UPD-175	Naphthoquinone	38.5 ± 6.9
UPD-176	Naphthoquinone	0.0 ± 0.2
UPD-182	Anthraquinone	46.9 ± 6.2
UPD-187	Anthraquinone	62.4 ± 10.0
UPD-196	Anthraquinone	89.6 ± 2.9
UPD-197	Anthraquinone	101.8 ± 1.6
UPD-200	Anthraquinone	118.7 ± 0.5
UPD-568	Anthracene	92.7 ± 11.4

UPD-576	Coumarin	105.2 ± 21.0
UPD-872	Naphthoquinone	118.8 ± 15.0
UPD-937	Indolin-dione	85.8 ± 7.8
UPD-1300	Quinolone	120.1 ± 0.7
UPD-1467	Quinone	111.7 ± 11.4

Table S2 - Enzymatic assay on CDC25A with compounds of the second round of selection

The compound concentration used was 20 μM. NSC-663284 was tested at concentration of 2 μM. The results are displayed as activity remaining (%) in the presence of compound relative to control reactions conducted in the presence of vehicle alone (DMSO). Values are averages of duplicate determinations ± standard deviations. One of two representative experiments is shown. Chemical structures were generated using 2D Sketcher, ChemDoodle®. Structures of compounds inhibiting CDC25A >90% are shown.

Compound	Structure	Class	% remaining activity ± SD
Ctrl		DMSO	100.0
NSC-663284	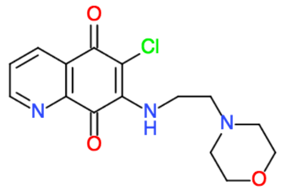	Quinolinedione	0.5 ± 0.6
UPD-140	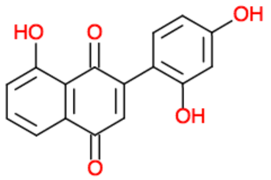	Naphthoquinone	0.1 ± 0.4
UPD-176	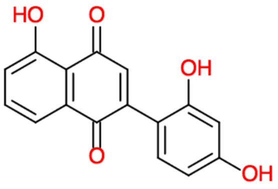	Naphthoquinone	0.0 ± 0.2

UPD-596	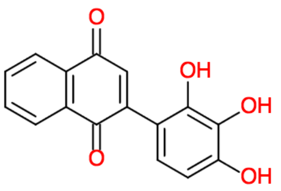	Naphthoquinone	0.0 ± 0.1
UPD-597	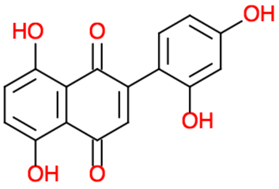	Naphthoquinone	3.1 ± 0.3
UPD-724	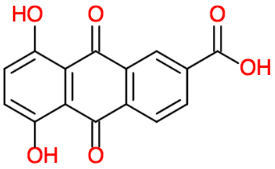	Anthraquinone	98.9 ± 1.4
UPD-738	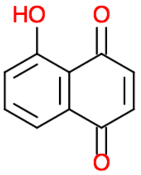	Naphthoquinone	5.9 ± 0.1
UPD-786	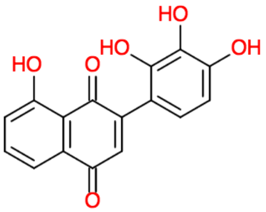	Naphthoquinone	0.0 ± 0.4
UPD-787	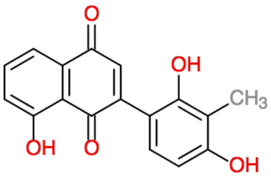	Naphthoquinone	0.1 ± 0.3
UPD-788	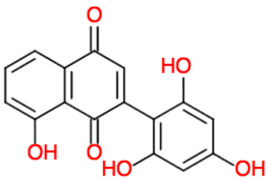	Naphthoquinone	70.1 ± 2.0

UPD-790	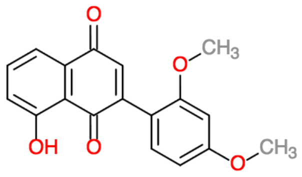	Naphthoquinone	5.6 ± 1.2
UPD-793	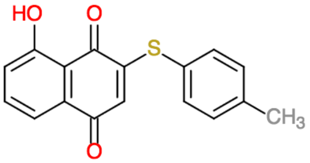	Naphthoquinone	1.0 ± 0.1
UPD-795	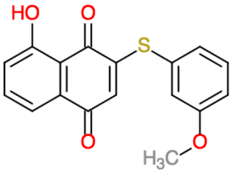	Naphthoquinone	0.0 ± 0.0
UPD-797	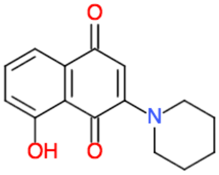	Naphthoquinone	8.5 ± 0.1
UPD-798	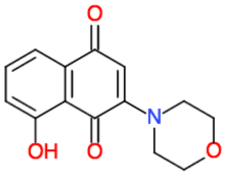	Naphthoquinone	41.4 ± 0.4
UPD-1382	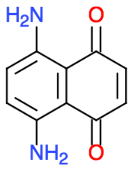	Naphthoquinone	94.4 ± 0.6
UPD-1416	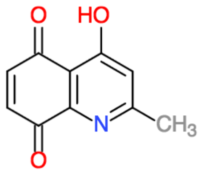	Quinone	4.1 ± 0.0
UPD-1419	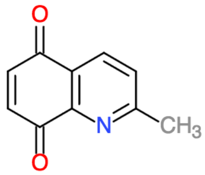	Quinone	1.9 ± 0.3

Table S3 - Kinetic parameters for UPD-795

The compound was incubated with CDC25 as described in Materials and Methods. Data were plotted using Prism software and kinetic parameters were calculated using non-linear regression of Michaelis-Menten curves with least-squares fit of the data and the mixed-model inhibition equation [8].

	**CDC25A**	**CDC25B**	**CDC25C**
K_i [µM]	1.18 ±0.4	0.44 ±0.1	0.85 ±0.3

Table S4 - Compound profiling

Enzymatic assays on selected phosphatases. The final concentration of UPD-795 and UPD-140 were 1 µM and 1.5 µM, respectively. Results are displayed as remaining activity (%) in the presence of compound relative to control reactions conducted in the presence of vehicle alone (DMSO). Values are averages of duplicate determinations ± standard deviation.

**Phosphatase**	**UPD-795**	**UPD-140**
Ctrl	100	100
PTPRC (CD45)	97 ± 2	103 ± 2
DUSP22	92 ± 3	90 ± 4
PTPN7 (HePTP)	87 ± 2	95
MKP5 (DUSP10)	107 ± 1	109
PP1α	88 ± 1	89 ± 2
PP2A	94 ± 1	95 ± 2
PP5	51 ± 6	43 ± 7
PTPN4 (MEG1)	94 ± 1	87 ± 1
PTPN9 (MEG2)	85 ± 5	89
PTPN1 (PTP1B)	94 ± 7	95 ± 5
PTPN22	98 ± 1	99 ± 5
PTPN6 (SHP-1)	80 ± 3	78 ± 2
PTPN11 (SHP-2)	91 ± 14	96 ± 2
PTPN2 (TCPTP)	99 ± 2	87 ± 8
TMDP (DUSP13)	100	105 ± 4
VHR (DUSP3)	108 ± 4	106 ± 4

SUPPLEMENTARY MOVIES

Movie M1 - Mitotic transition in control-treated Kyoto HeLa cells

Time course visualization of mitotic transition in Kyoto HeLa cells treated with vehicle and visualized as described in Fig. S7.

Movie M2 - Mitotic transition in UPD-787-treated Kyoto HeLa cells

Time course visualization of mitotic transition in Kyoto HeLa cells treated with UPD-787 (5 μM) and visualized as described in Fig. S7.

Movie M3 - Mitotic transition in UPD-790-treated Kyoto HeLa cells

Time course visualization of mitotic transition in Kyoto HeLa cells treated with UPD-790 (5 μM) and visualized as described in Fig. S7.

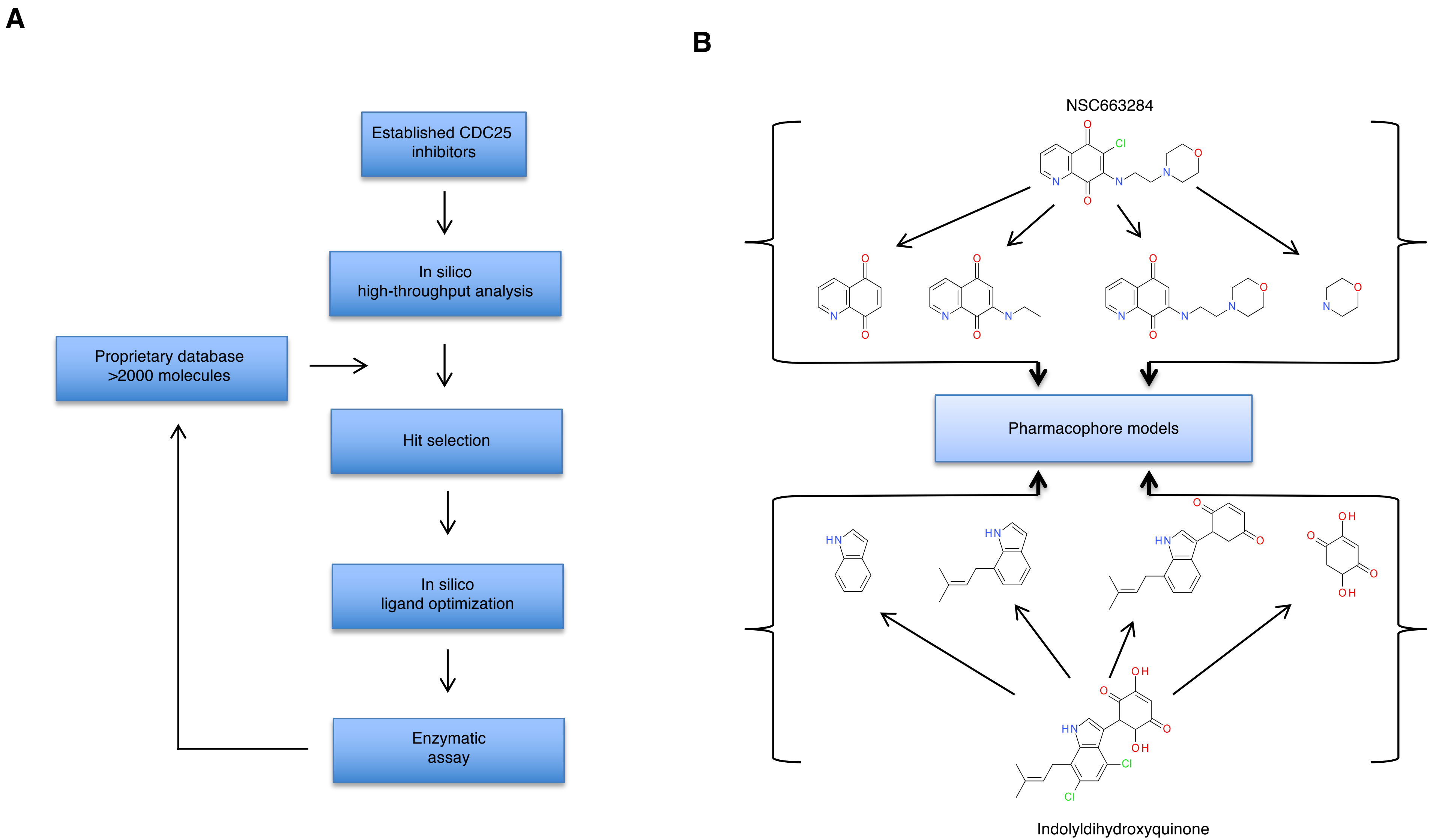
A
Established CDC25 inhibitors
In silico high-throughput analysis
Proprietary database >2000 molecules
Hit selection
In silico ligand optimization
Enzymatic assay
B
NSC663284
Pharmacophore models
Indolyldihydroxyquinone

Figure S1

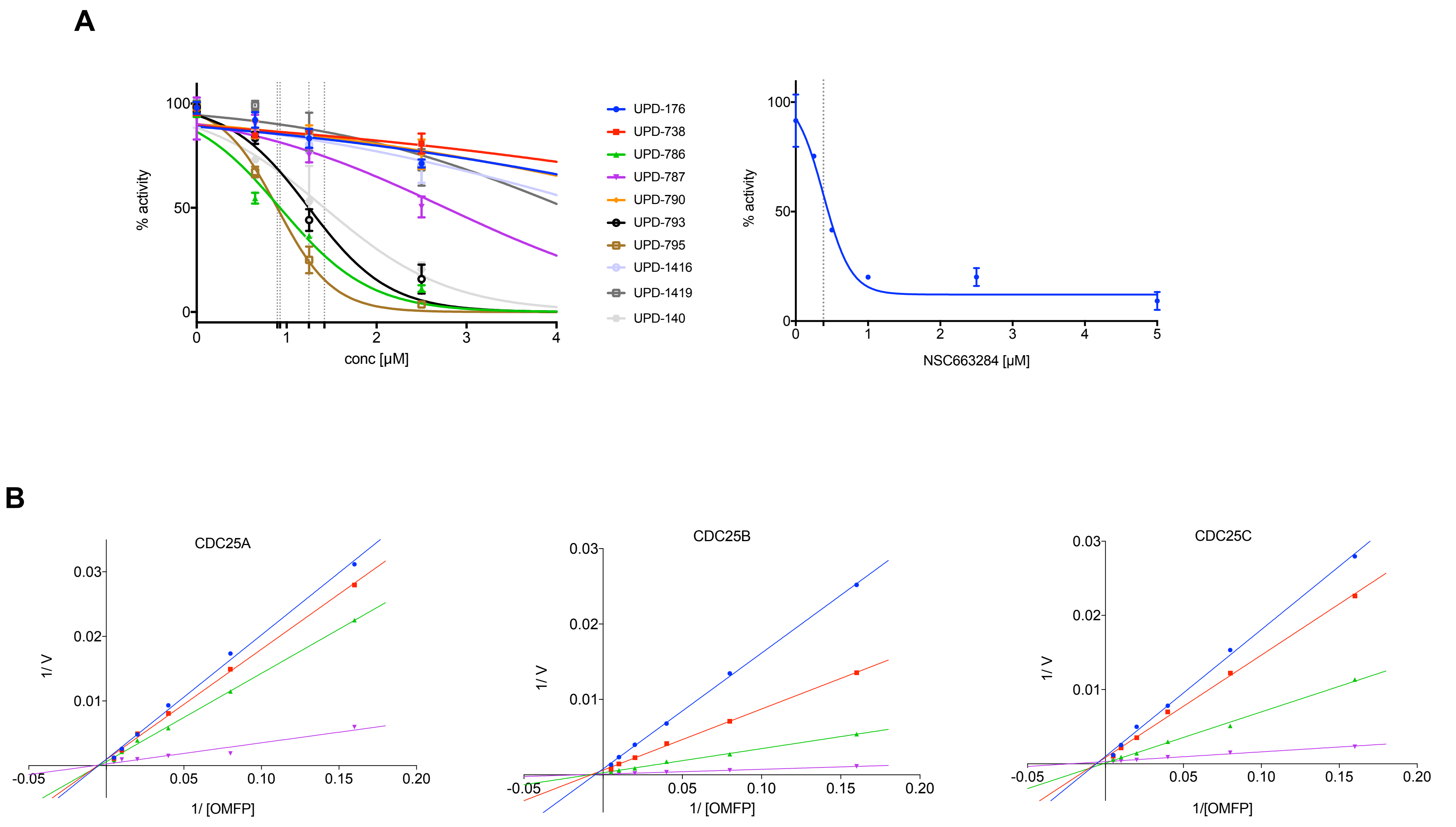

A
100
50
0
% activity
0
1
2
3
4
conc [μM]
UPD-176
UPD-738
UPD-786
UPD-787
UPD-790
UPD-793
UPD-795
UPD-1416
UPD-1419
UPD-140
% activity
NSC663284 [μM]
5
B
CDC25A
CDC25B
CDC25C
1/ V
0.03
0.02
0.01
-0.05
0.05
0.10
0.15
0.20
1/ [OMFP]
1/[OMFP]

Figure S2

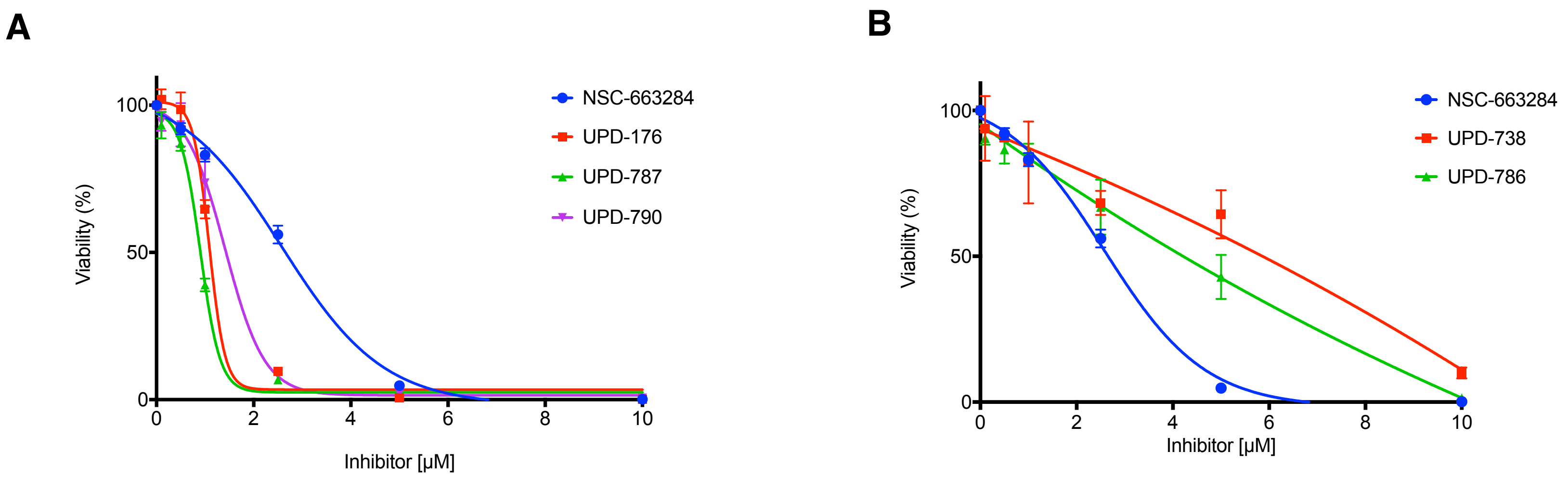
A
Viability (%)
100
50
0
0
2
4
6
8
10
Inhibitor [μM]
NSC-663284
UPD-176
UPD-787
UPD-790
B
Viability (%)
100
50
0
0
2
4
6
8
10
Inhibitor [μM]
NSC-663284
UPD-738
UPD-786

Figure S3

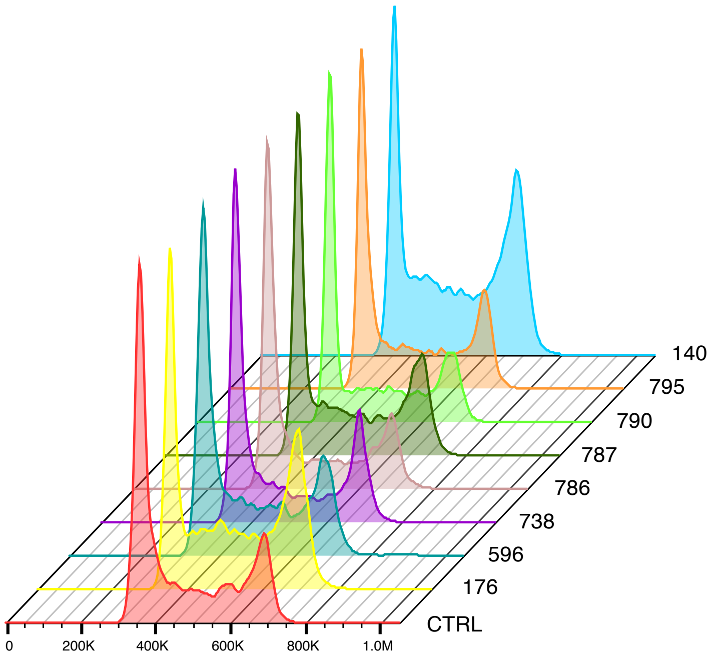

Figure S4

A

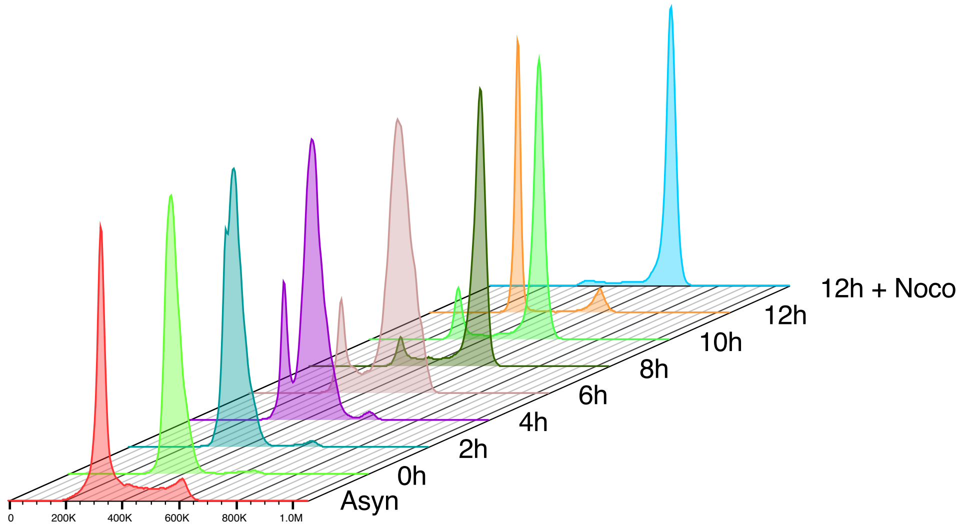

12h + Noco
12h
10h
8h
6h
4h
2h
0h
Asyn
0
200K
400K
600K
800K
1.0M

B

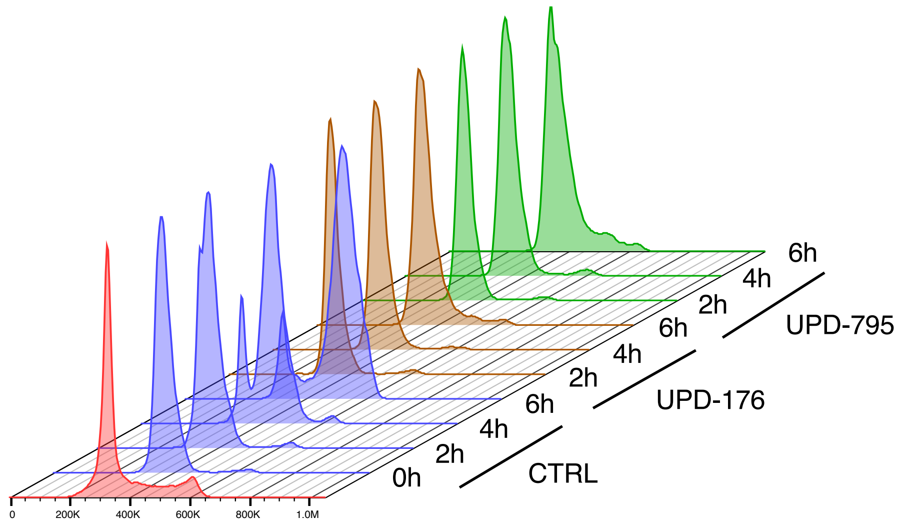

6h
4h
2h
UPD-795
6h
4h
2h
UPD-176
6h
4h
2h
0h
CTRL
0
200K
400K
600K
800K
1.0M

Figure S5

A

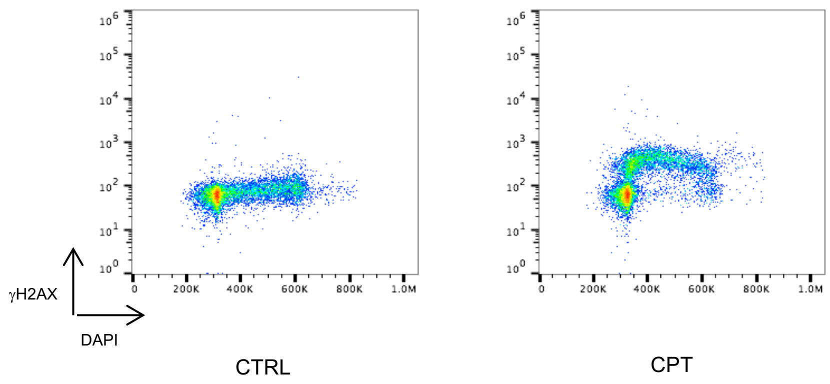

10^6
10^5
10^4
10^3
10^2
10^1
10^0
0
200K
400K
600K
800K
1.0M
γH2AX
DAPI
CTRL
CPT

B

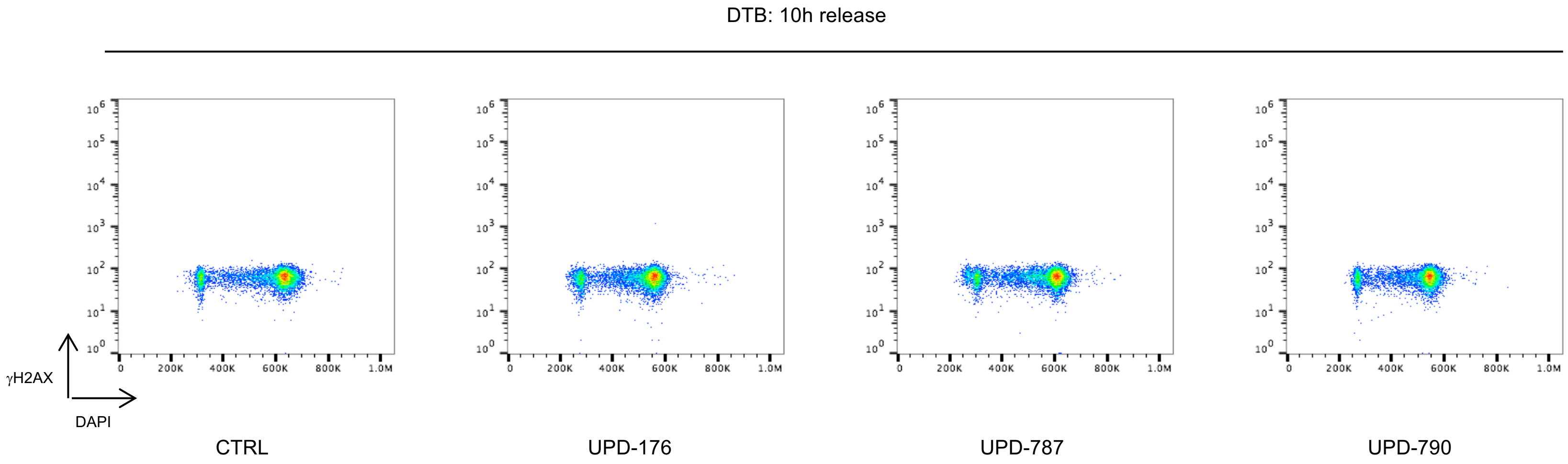

DTB: 10h release
10^6
10^5
10^4
10^3
10^2
10^1
10^0
0
200K
400K
600K
800K
1.0M
γH2AX
DAPI
CTRL
UPD-176
UPD-787
UPD-790

Figure S6

A

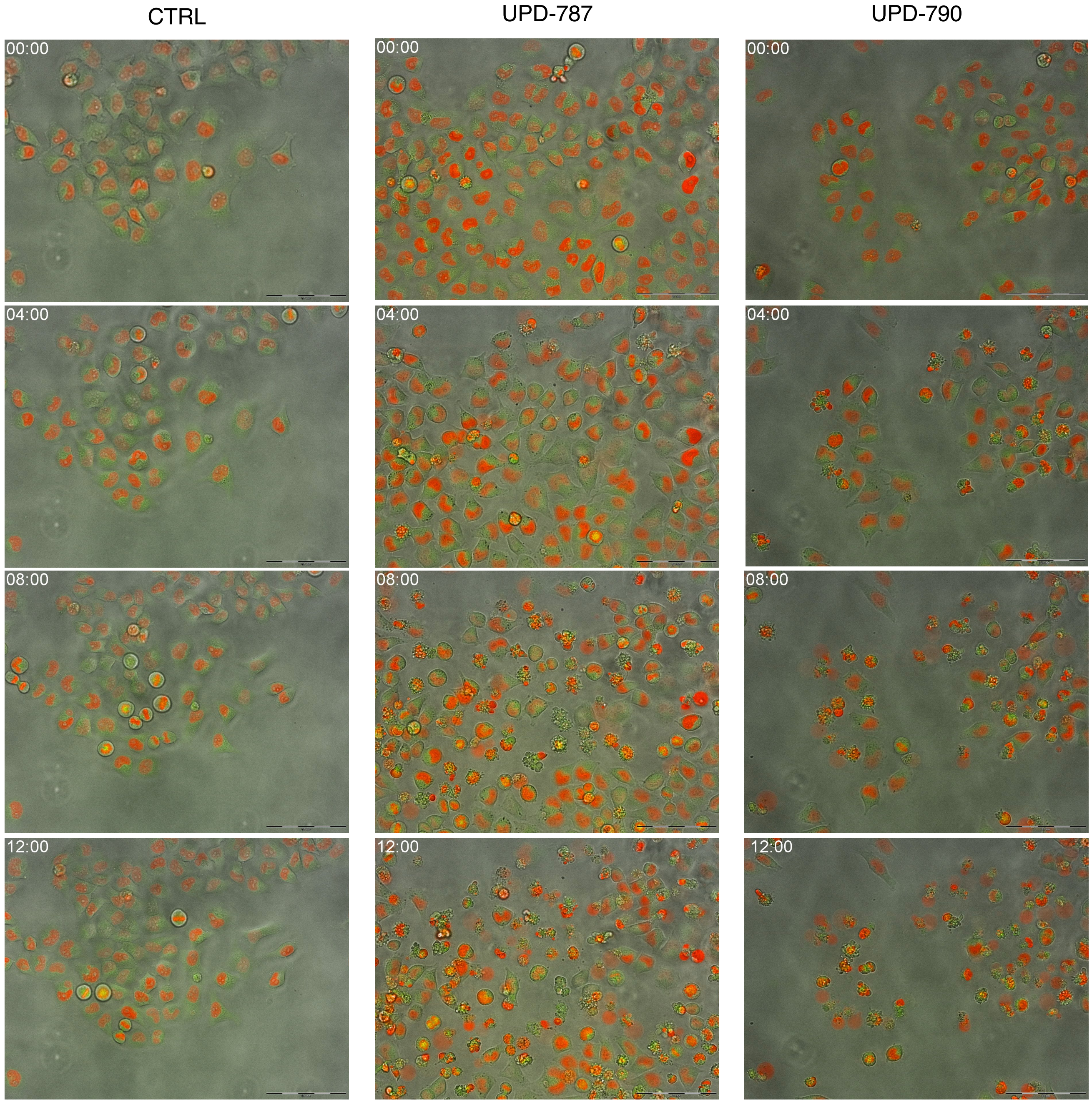
CTRL
UPD-787
UPD-790
00:00
04:00
08:00
12:00

B

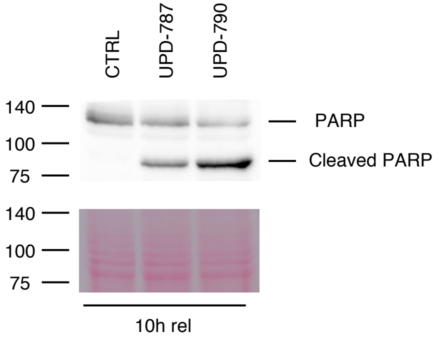
CTRL
UPD-787
UPD-790
140
100
75
PARP
Cleaved PARP
140
100
75
10h rel

Figure S7

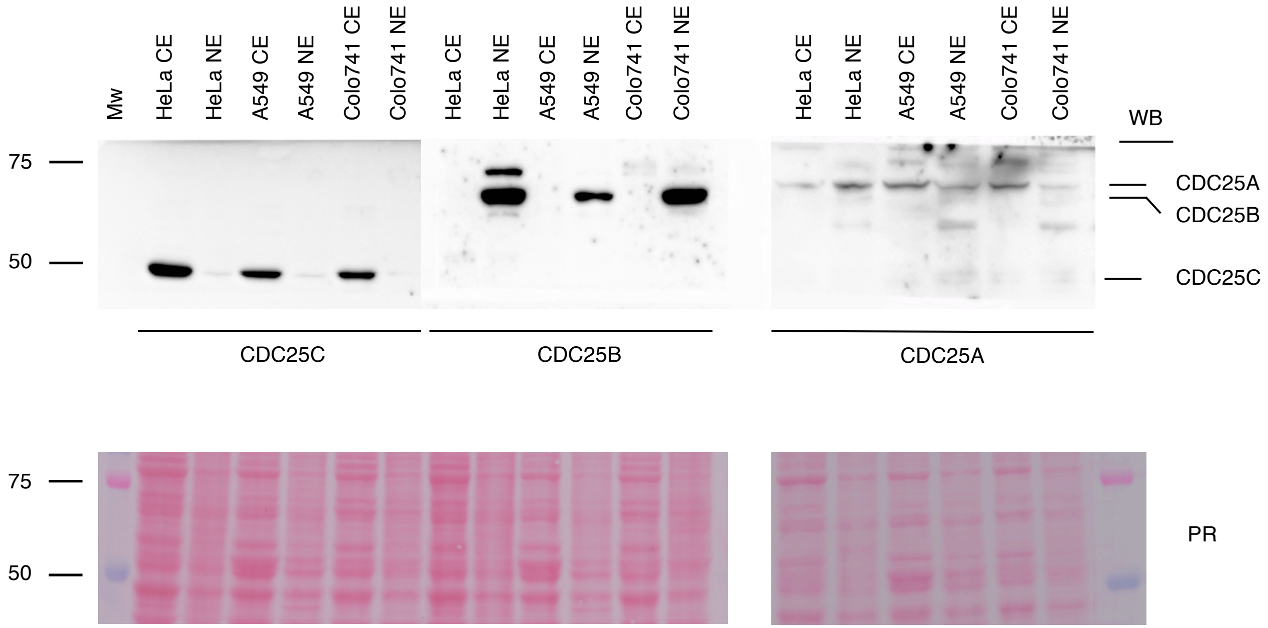
Mw
HeLa CE
HeLa NE
A549 CE
A549 NE
Colo741 CE
Colo741 NE
HeLa CE
HeLa NE
A549 CE
A549 NE
Colo741 CE
Colo741 NE
HeLa CE
HeLa NE
A549 CE
A549 NE
Colo741 CE
Colo741 NE
WB
75
50
CDC25A
CDC25B
CDC25C
CDC25C
CDC25B
CDC25A
75
50
PR

Figure S8

Nonlin fit<br>Table of results		A<br>176 Cdc25A^{+}	B<br>176 Cdc25A^{-}	C<br>Global (shared)
		Y	Y	Y
1	**Comparison of Fits**			
2	Null hypothesis			One curve for all data sets
3	Alternative hypothesis			Different curve for each data set
4	P value			<0.0001
5	Conclusion (alpha = 0.05)			Reject null hypothesis
6	Preferred model			Different curve for each data set
7	F (DFn, DFd)			11.90 (4, 30)
8				
9	**Different curve for each data set**			
10	**Best-fit values**			
11	Bottom	0.8187	4.684	
12	Top	145.7	112.6	
13	LogIC50	1.359	1.460	
14	HillSlope	-0.2684	-0.7443	
15	IC50	22.86	28.81	
16	Span	144.9	107.9	
17	**Std. Error**			
18	Bottom	2.513	2.339	
19	Top	20.56	6.748	
20	LogIC50	0.4879	0.1231	
21	HillSlope	0.04193	0.1308	
22	Span	21.87	7.568	

Nonlin fit<br>Table of results		A<br>787 Cdc25A^{+}	B<br>787 Cdc25A^{-}	C<br>Global (shared)
		Y	Y	Y
1	**Comparison of Fits**			
2	Null hypothesis			One curve for all data sets
3	Alternative hypothesis			Different curve for each data set
4	P value			<0.0001
5	Conclusion (alpha = 0.05)			Reject null hypothesis
6	Preferred model			Different curve for each data set
7	F (DFn, DFd)			37.39 (4, 34)
8				
9	**Different curve for each data set**			
10	**Best-fit values**			
11	Bottom	-0.4542	2.256	
12	Top	134.8	137.4	
13	LogIC50	1.873	0.8877	
14	HillSlope	-0.2748	-0.5184	
15	IC50	74.68	7.722	
16	Span	135.2	135.1	
17	**Std. Error**			
18	Bottom	3.348	1.754	
19	Top	18.00	15.56	
20	LogIC50	0.4672	0.2176	
21	HillSlope	0.05121	0.07094	
22	Span	19.73	16.21	

Figure S9

Fig 4B_top

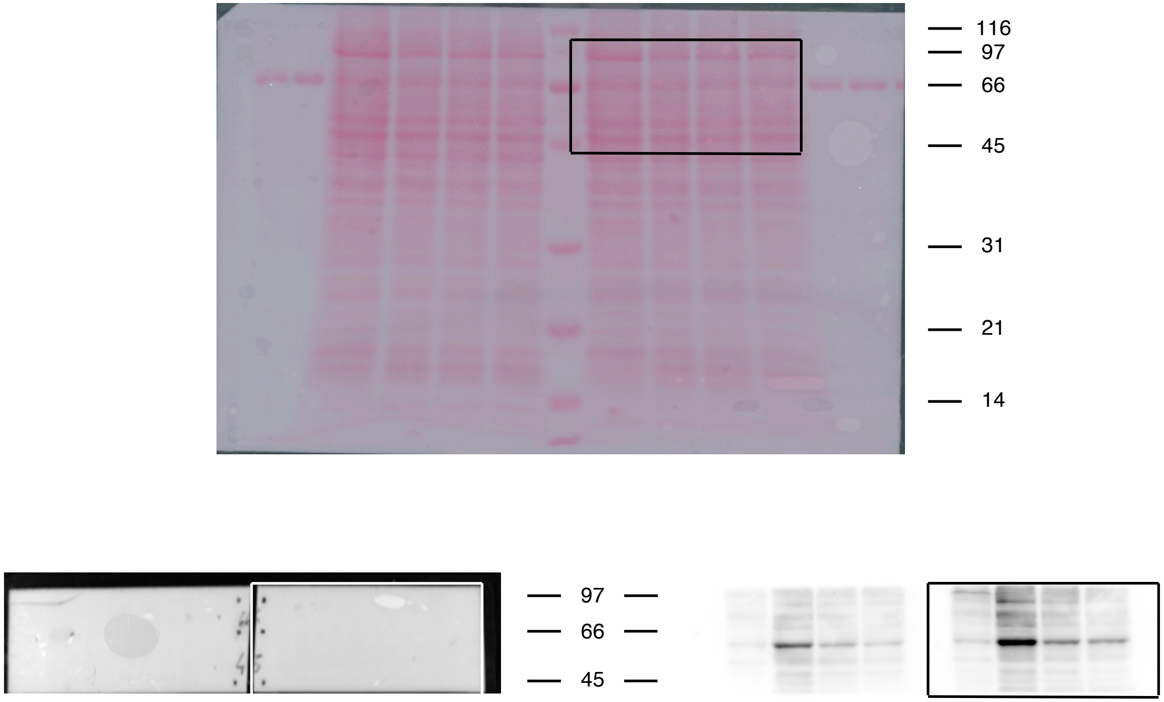

50 ug cell extracts on 12% gel;
Primary: MPM2 monoclonal (Upstate) 1:1000 in 2% BSA
Secondary: anti mouse HRP 1:5000 in 2% BSA

Fig 4B_bottom

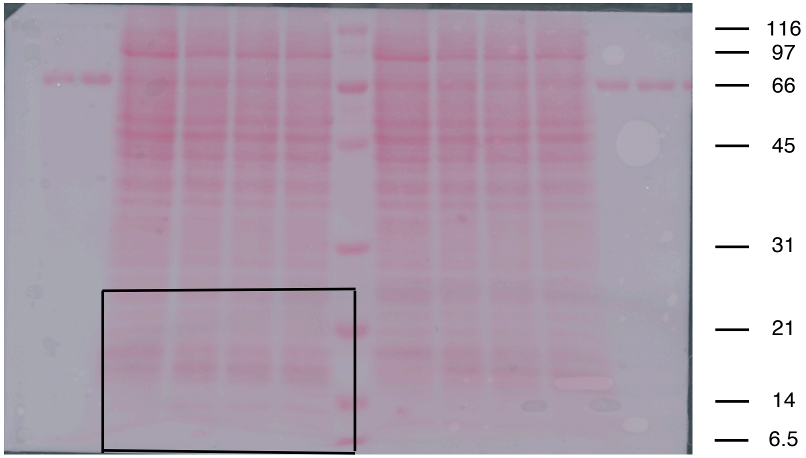

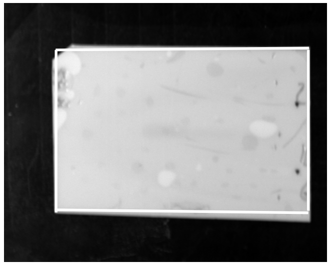

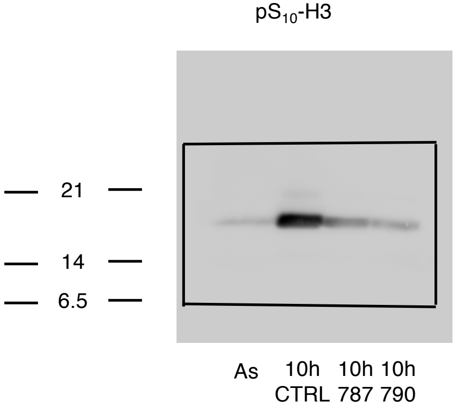

50 ug HeLa extracts on 12% gel
Primary antibody: rabbit anti-pH3-S10 (CST) 1:1000 in 2% BSA
Secondary antibody: anti-rabbit HRP 1:5000 in 2% BSA

Fig 4C

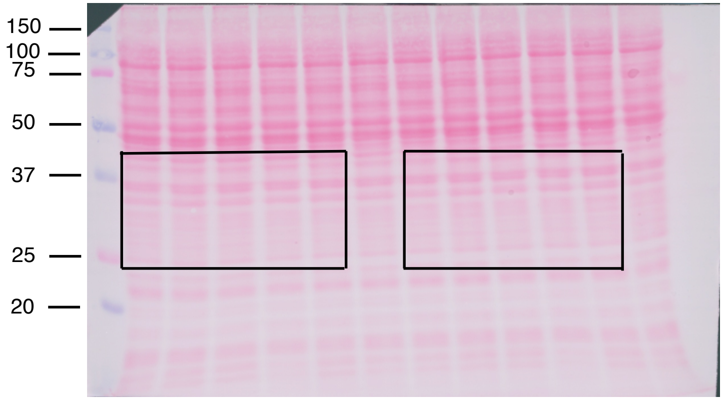

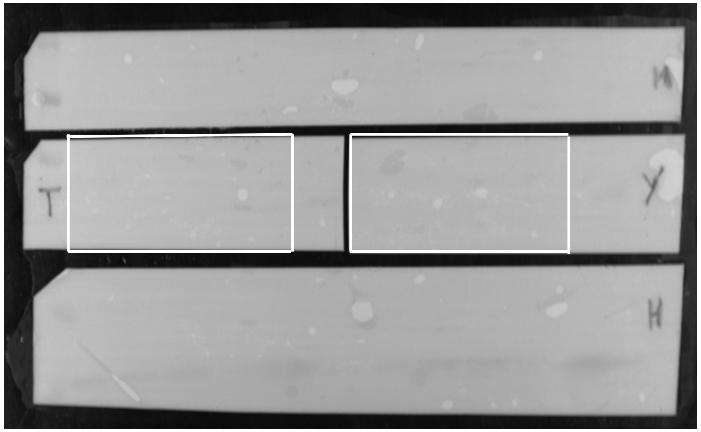

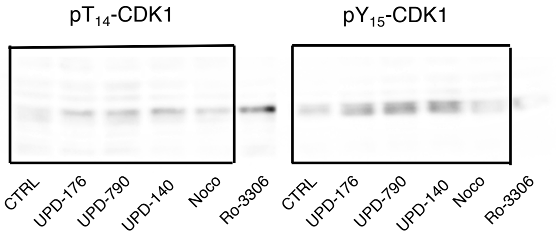

100 ug HeLa extracts on 12% gel
Primary antibody: rabbit anti-pCDK1-T14, rabbit anti pCDK1-Y15 (CST) 1:1000 in 2% BSA
Secondary antibody: anti-rabbit HRP 1:5000 in 2% BSA

Fig 5B

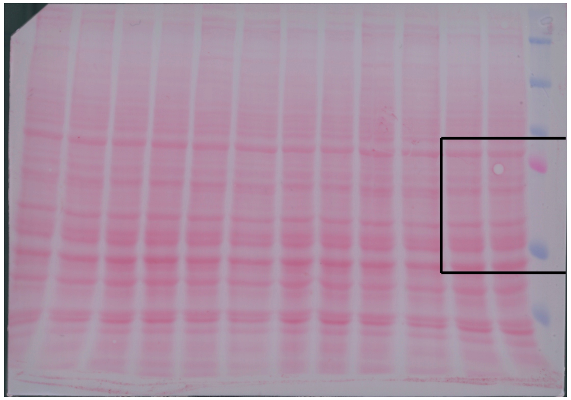

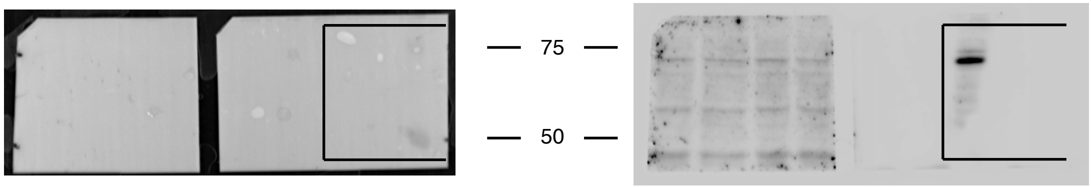

50 ug cell extracts on 10% gel;
Primary: HA rabbit polyclonal (Abcam) 1:1000 in 2% BSA
Secondary: anti rabbit HRP 1:5000 in 2% BSA

Fig S8_left

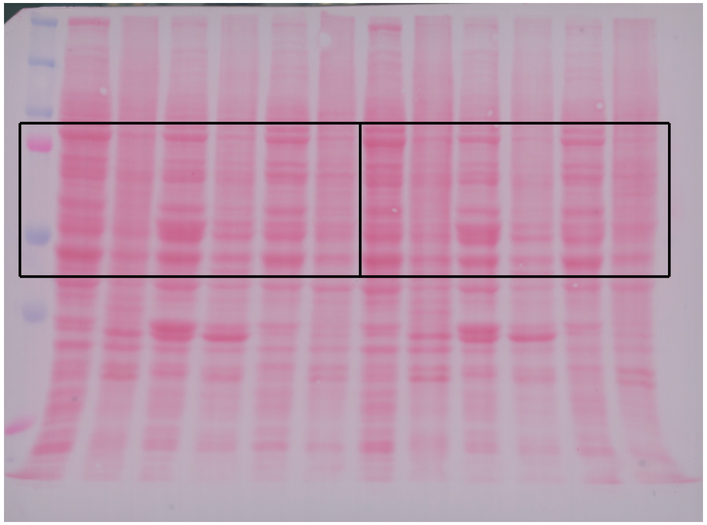

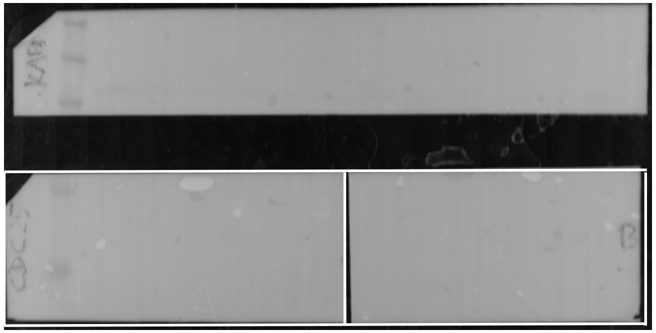

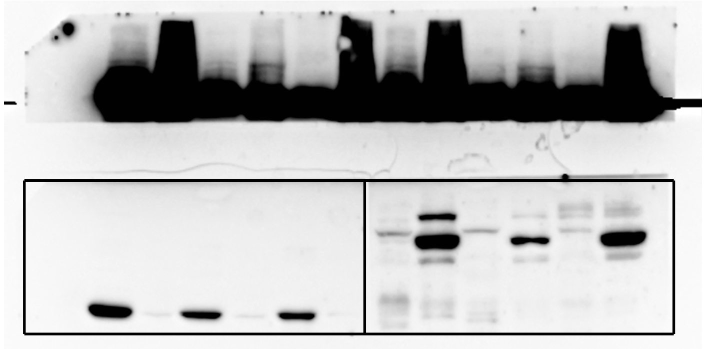

CDC25C1 = 53365
CDC25C2 = 50122
CDC25C4 = 48851

CDC25B1 = 64987
CDC25B2 = 60756

100 ug cell extracts on 10% gel
Primary: CDC25C rabbit polyclonal (S.Cruz C-20) 1:100 in 2.5% milk; CDC25B rabbit monoclonal (CST) 1:500 in 2.5% milk
Secondary: anti-rabbit 1:5000 in 2.5% milk

Fig S8_right

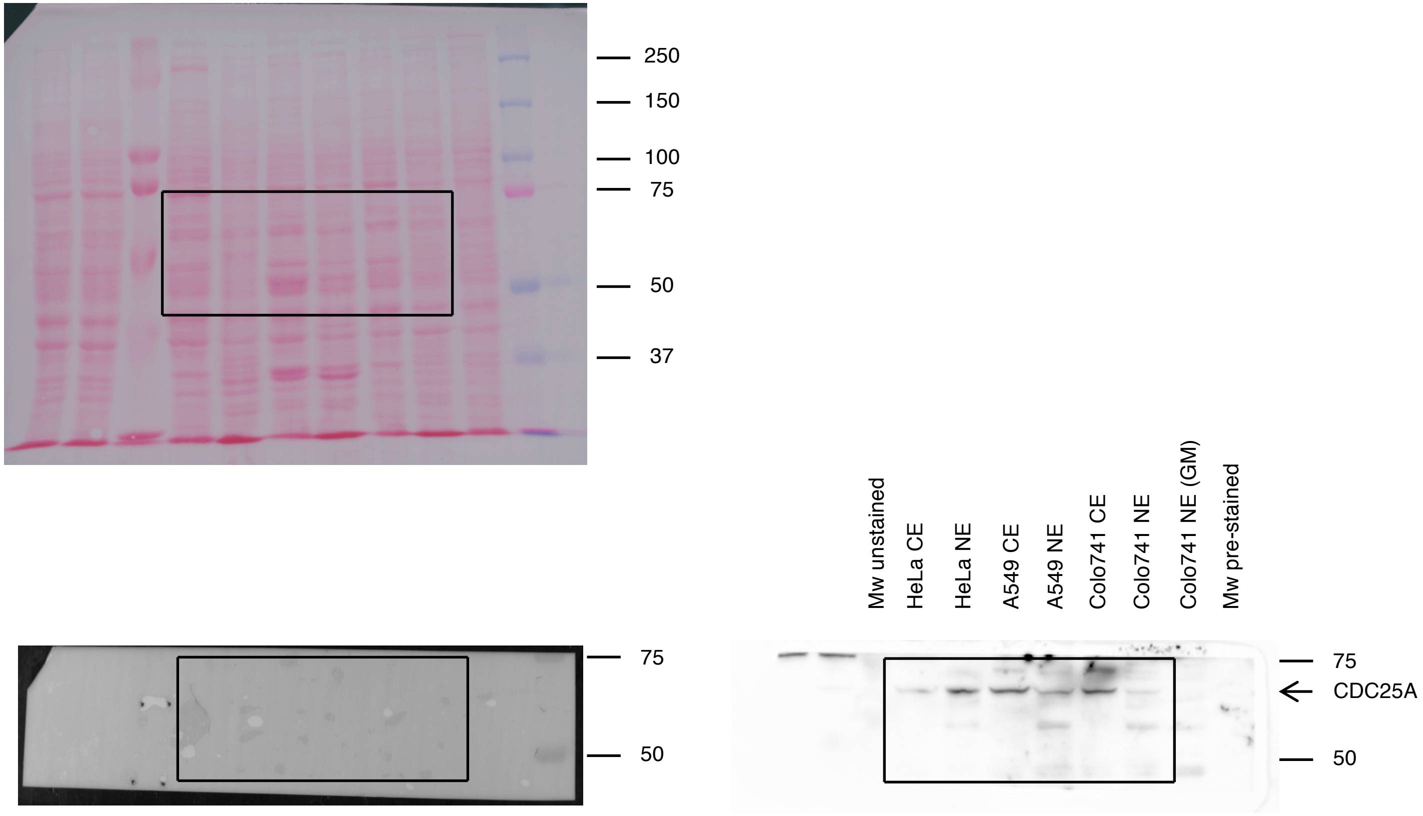

30 ug cell extracts on 8% gel
Primary: CDC25A monoclonal F6 1:250 in 5% milk
Secondary: anti-mouse IgG-k 1:5000 in milk
